# A Competition between Relative Stability and Binding Energy in Caffeine Phenyl-Glucose Aggregates: Implications in Biological Mechanisms

**DOI:** 10.3390/ijms24054390

**Published:** 2023-02-23

**Authors:** Camilla Calabrese, Ander Camiruaga, Maider Parra-Santamaria, Luca Evangelisti, Sonia Melandri, Assimo Maris, Imanol Usabiaga, José A. Fernandez

**Affiliations:** 1 Departamento de Química Física y Química Inorgánica, Facultad de Ciencias—I.U. CINQUIMA, Universidad de Valladolid, E-47011 Valladolid, Spain; 2 Institut des Sciences Moléculaires d’Orsay (ISMO), UMR8214, Université Paris-Saclay, CNRS, Bat. 520, F-91405 Orsay, France; 3 Departamento de Química Física, Facultad de Ciencias y Tecnología, Universidad del País Vasco (UPV/EHU), Barrio Sarriena s/N, E-48940 Leioa, Spain; 4 Dipartimento di Chimica “Giacomo Ciamician”, Campus of Ravenna, Università di Bologna, Via Sant’Alberto, 163, 48123 Ravenna, Italy; 5 Dipartimento di Chimica “Giacomo Ciamician”, Università di Bologna, Via Selmi, 2, 40126 Bologna, Italy

**Keywords:** caffeine, sugars, quantum mechanical calculations, UV/IR spectroscopy, noncovalent interactions

## Abstract

Hydrogen bonds and stacking interactions are pivotal in biological mechanisms, although their proper characterisation within a molecular complex remains a difficult task. We used quantum mechanical calculations to characterise the complex between caffeine and phenyl-β-D-glucopyranoside, in which several functional groups of the sugar derivative compete with each other to attract caffeine. Calculations at different levels of theory (M06-2X/6-311++G(d,p) and B3LYP-ED=GD3BJ/def2TZVP) agree to predict several structures similar in stability (relative energy) but with different affinity (binding energy). These computational results were experimentally verified by laser infrared spectroscopy, through which the caffeine·phenyl-β-D-glucopyranoside complex was identified in an isolated environment, produced under supersonic expansion conditions. The experimental observations correlate with the computational results. Caffeine shows intermolecular interaction preferences that combine both hydrogen bonding and stacking interactions. This dual behaviour had already been observed with phenol, and now with phenyl-β-D-glucopyranoside, it is confirmed and maximised. In fact, the size of the complex’s counterparts affects the maximisation of the intermolecular bond strength because of the conformational adaptability given by the stacking interaction. Comparison with the binding of caffeine within the orthosteric site of the A2A adenosine receptor shows that the more strongly bound caffeine·phenyl-β-D-glucopyranoside conformer mimics the interactions occurring within the receptor.

## 1. Introduction

Caffeine (3,7-dihydro-1,3,7-trimethyl-1H-purine-2,6-dione, Caf hereafter) is a naturally occurring alkaloid, the main member of the methylated xanthine family [1]. Caf is a rigid and planar molecule, capable of producing very stable stacking interactions [2], and due to its similarity to the purinic RNA/DNA bases, it can interfere with the metabolic system [3]. This stimulating substance, commonly present in high concentrations in coffee and many other popular energetic drinks, acts as a nonselective antagonist of the adenosine receptors (A_1_, A_2A_, A_2B_ and A_3_) in the human brain [4,5,6]. This biochemical behaviour gives Caf its well-known effects, allowing prolongation of the daily active period [7]. Inside the orthosteric binding pockets of the A_2A_ adenosine receptor, Caf primarily interacts with phenylalanine (F168) and asparagine (N253) residues, via stacking interactions and hydrogen bonds, respectively [8,9]. However, the specific chemical variables responsible of the molecular mechanisms underlying the effects of Caf have not yet been isolated and fully identified.

**Figure 1:**
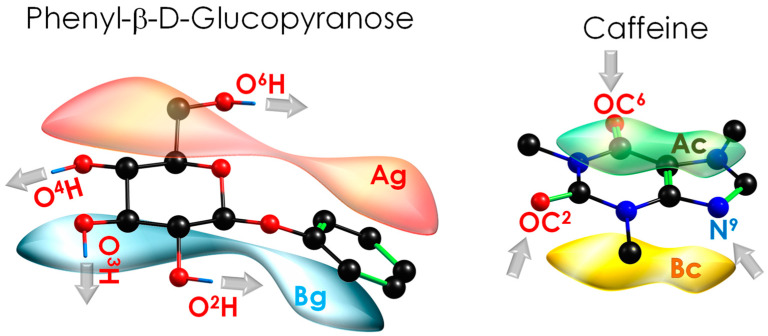
Phenyl-β-D-glucopyranoside (PhGlc) and caffeine (Caf) structures, with main binding sites highlighted. Note that the absence of symmetry in PhGlc results in several stacking orientations for the complex: the “A” surface of phenyl glucose (depicted in red, Ag) can interact with the “A” surface of caffeine (depicted in green, Ac) producing AgAc stacking structure. In the same way, other three combination are possible: AgBc, BgAc and BgBc, where the “B” surface of phenyl glucose (depicted in blue, Bg) and B surface of caffeine (depicted in yellow, Bc) combine with each other.

Stacking interactions mediated by aromatic rings are crucial in docking processes inside receptors [10,11,12,13]. Characterisation of the interaction preferences of biomolecules, such as Caf, helps to understand the mechanisms that rule the docking processes. In fact, purine derivatives perform many important functions in human metabolism, and recent studies report the preferences of Caf for establishing stacking interactions with saccharides [14]. To investigate the stacking preferences of Caf and isolate the key chemical contacts related to its biological activity, a specific binding partner has been selected: phenyl-β-D-glucopyranoside (PhGlc). Caf presents an aromatic heterocyclic fused ring prone to form stacking interactions, as well as four nitrogen atoms and two carbonyl oxygens that may serve as anchor points for the intermolecular interactions. On the other hand, PhGlc presents a set of flexible hydroxyl groups coordinated through a hydrogen bond network that extends along the carbon backbone (Figure 1).

In this work, we will deepen the characterisation of the intermolecular forces inside Caf and other biological functional groups, mainly phenol and glucose. For this project, we make use of extended computational analysis to calculate the surface of intermolecular potential energy (iPES), as accomplished in the previous work for Caf and phenol (Ph) complex [15]. The validated computational procedure used for the Caf+Ph complex is here expanded to the case of Caf+PhGlc. The flexibility of PhGlc in comparison with the Ph ligand demands a more accurate classification of the possible structural minima. The conformational panorama of the interactions between these two molecules reveals some general trends: there are two more stable families of conformations. The most stable one presents the PhGlc in its most stable arrangement, and Caf adapts its position to maximise the intermolecular interaction. On the other hand, there is the family of structures where PhGlc adopts a less stable conformation in favour of producing a stronger interaction with Caf. This is a rarely observed effect: two families are similar in terms of relative stability, but in one of these glucose produces stronger intramolecular interactions and weaker intermolecular interactions, while in the second family, the increase of the intramolecular hindrance gives rise to a more efficient intermolecular interaction. Both families are similar in terms of overall stability, but in one case the energetically optimised section is the intramolecular part while in the other case it is the intermolecular part.

Finally, to experimentally prove the computational work, we perform mass-resolved laser infrared spectroscopy under supersonic expansion conditions. These isolated experimental conditions are one of the most appropriate to be compared with quantum chemical calculations. The collisional cooling produced during the expansion enables the formation of a rich collection of molecular aggregates that travel cooled (vibrational temperature of 10–200 K) and isolated, forming a dense beam of species in the high vacuum of the ionisation chamber of a mass spectrometer (usually a time-of-flight mass spectrometer, namely TOF). A number of sophisticated laser-based spectroscopic techniques have been developed to probe the beam, measuring different observables from the species formed. The use of mass-resolved detection allows also to discriminate between aggregates of different stoichiometry, while the high-resolution power of the spectroscopic techniques may isolate the contribution from the conformational isomers of a given stoichiometry. For these reasons, the mass-resolved and conformer-selective laser spectroscopy in supersonic expansions is the best tool to support computational chemistry methods in the study of noncovalent interactions. Many systems have been tackled in this way, ranging from very small van der Waals complexes to nanometre-sized ones [16,17].

## 2. Results

### 2.1. Exploration of the Intermolecular Potential Energy Surface (iPES)

Previous studies on Caf+Phenol [15] and Caf+Water [18] have already reported the complexity of these systems, especially when modelling and isolating the rich collection of local minima in iPES. The study of the present system is a step forward in complexity because of the relatively large size and flexibility of PhGlc compared to that of phenol or water. The Caf+Phenol [15] study demonstrated that many standard computational methods are not accurate in modelling complexes and/or vibrational frequencies, in which the final shapes are determined by a subtle balance between several weak interactions. In this study, we used two computational methods to test the validity of the computational predictions: a Minnesota functional [19], which has proven to be appropriate for the description of sugars (M06-2X/6-311++G(d,p)) [20,21,22,23], along with Grimme’s dispersion-corrected B3LYP functional (B3LYP-GD3BJ) coupled to the def2TZVP basis set, which has recently demonstrated to produce accurate predictions for the spectra recorded in jets [24,25,26,27].

**Figure 2:**
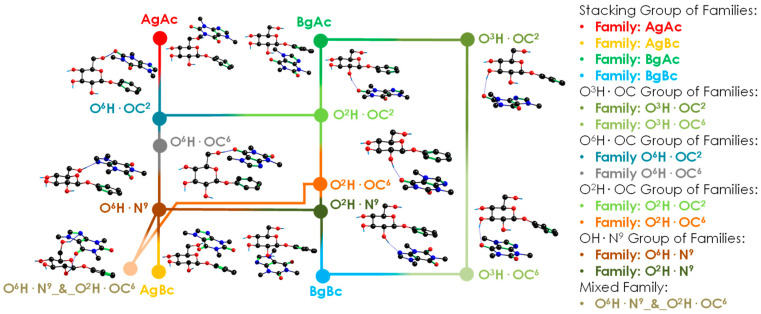
Here a simplified picture of the iPES is shown. There are 13 families or interaction’s types identified. These interactions are classified in groups of families that are related to the similarity in the interaction and the related infrared spectral contribution. The average spectral contributions of each family are available in Table S1, while the contributions of each group of families are presented in Figure S1.

**Figure 3:**
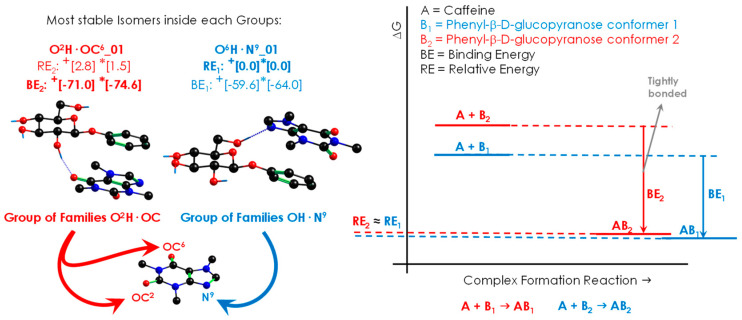
Most stable structures of Caf+PhGlc. Energy values (kJ/mol) are given for the two different computational methods used: (+) M06-2X/6-311++G(d,p) and (*) B3LYP-GD3BJ/def2TZVP. The first couple of values for each method refers to the relative energy (RE) while the second one is the binding energy (BE), calculated at 0 K. RE_X_ refers to the energy of the complex X minus the energy of the most stable complex. BE_X_ refers to the energy of the complex X minus the energy of the sum of the monomers in the most similar arrangement as present in the complex. The label of each structure uses as reference the main interaction forming the complex. The label of the most stable (RE: O^6^H·N^9^_01) and that of the most strongly bonded structures (BE: O^2^H·OC^6^_01 with O^2^H·OC^2^_01) are in bold.

An extract of the extensive theoretical work is summarised in Figure 2. The 108 minimised isomers from the conformational search were classified into 13 families, connected by many isomerisation pathways (see Figure S1). The structures were classified according to the orientation of the two molecules with direct π···π interaction or stacking (Ag, Bg, Ac and Bc, see Figure 1) and to the functional groups that form hydrogen bonds (O^6^H, O^2^H, and O^3^H for PhGlc; OC^2^, OC^6^, and N^9^ for Caf). Thus, most of the conformers present a stacking interaction plus a hydrogen bond between one of those moieties. Figure 3 shows the most stable conformations, together with their relative energy (RE, the global minimum is highlighted in blue) and the highest binding energy (BE, highlighted in red). The complete set of structures, together with the analysis of each family, is reported in the Supplementary Material (Figures S2–S23). Moreover, in order to rationalise the structures, we classified and organised the complexes according to their RE and BE and the specific interactions within each family and group of families (see Figures S24 and S25), where it can be noted that the energy trend is maintained both by considering the minima of each family and by taking the average of the conformers belonging to each family (Figure S25).

The rich collection of interactions displayed on the iPES results in a group of structures with similar relative energies but with substantial differences in the binding strengths. The flexibility of PhGlc is the reason behind this difference, which leads to two possible scenarios: (i) a very stable conformation of the sugar gives rise to a less effective interaction with Caf, (ii) PhGlc adopts a less stable arrangement, entailing an increase in binding energy. This phenomenon causes a mismatch between the most stable structures (lower RE) and the most tightly bonded structures (higher BE), as shown in Figure 3.

The structures reported in Figure 2 belong to different groups of families, which are close in stability: OH·N^9^ and O^2^H·OC. One of them, O^2^H·OC, is characterised by a stronger binding energy with respect to the rest (highlighted in red). To evaluate the reliability of this energetic trend, the most stable structures of each family were also optimised using other commonly used levels of theory: B3LYP-GD3BJ/6-311++G(d,p), B97D/6-311++G(d,p) and ωB97X/6-311++G(d,p). The energy data obtained are summarised in Figures S24 and S26, where the binding Gibbs-free energy at 0 K and 298 K are also shown. It is worth noting the considerable gap in relative stability (around 9 kJ/mol) between the red group (O^2^H·OC) and the other ones. Interestingly, the general trend is also maintained at high temperature: the orange family is still the most strongly bonded. The stronger binding energy is an important feature and can be used to extrapolate the aggregation behaviour to the condensed phase. Even if the latter is affected by the medium, this trend could represent a key point during the selective docking in biological processes.

### 2.2. Spectroscopic Characterisation of Caf+PhGlc Complex

The Caf+PhGlc adduct presents not only a complex conformational landscape but also a difficult spectroscopy to analyse. Transferring these compounds into the isolated phase requires a sophisticated experimental setup, such as laser desorption systems. In addition, an inefficient cooling process over the low-frequency vibrational modes of a complex and the low efficiency in the excitation processes result in a lower signal-to-noise ratio. The REMPI spectrum of Caf+PhGlc may be found in Figures S27 and S28. A broad and unstructured absorption spectrum was obtained for the complex, in clear contrast with those obtained from the monomers. The chromophore in PhGlc was employed to excite the dimer, as it produced more signal. The most valuable structural information was extracted from the IR/UV double resonance technique. Two excitation wavelengths were probed in order to investigate different conformations, but they both showed a similar spectrum.

**Figure 4:**
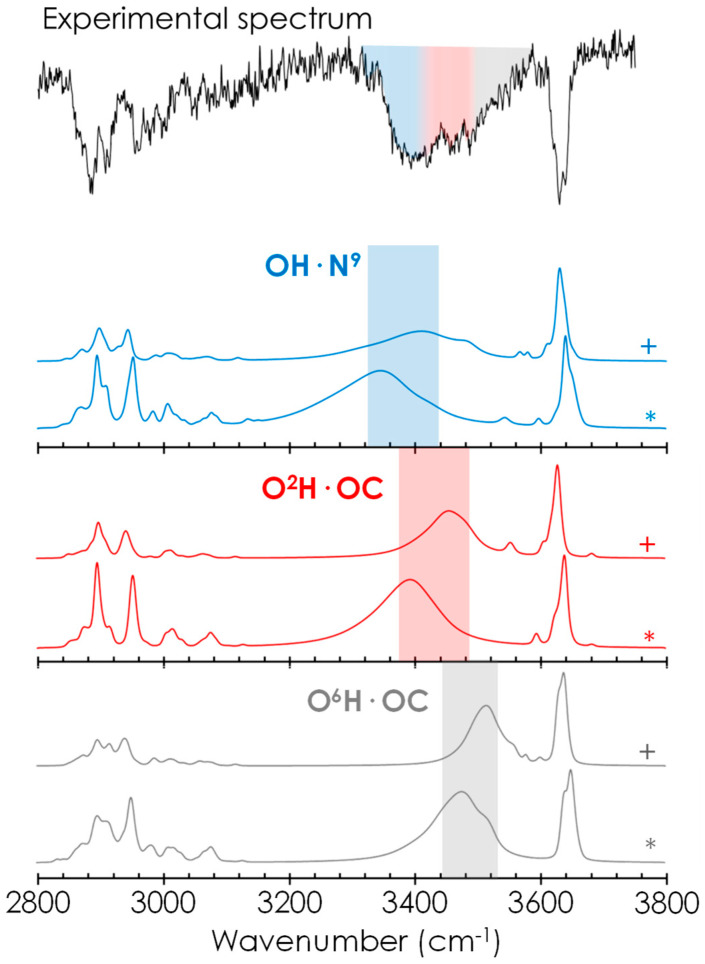
Above the experimental spectrum, below the computation average simulation for the three groups of families with absorption in the experimental observed range. The theoretical simulation at M06-2X/6-311++G(d,p) level are labelled with (+) and the theoretical simulation at B3LYP-GD3BJ/def2TZVP level are labelled with (*).

Confirmation of the propensity of the system to form the most strongly bonded structures comes from the experimental mass-resolved conformer-selective IR spectrum (Figure 4 and Figure S27). The experimental trace is composed of: (i) a group of unresolved bands at ~3610 cm^−1^ due to the stretching vibrations of free or intramolecularly bonded glucose hydroxyl groups, (ii) a broad absorption that extends from 3300 to 3590 cm^−1^ and that very likely comprises the contribution of several OH moieties involved in the formation of hydrogen bonds of variable strength and (iii) a complex group of transitions between 2800 and 3100 cm^−1^ due to CH stretching vibrations. The broad nature of the experimental spectrum prevents the traditional assignment of the spectrum by combination of several isomers; in this case we cannot speak of a particular isomer because we do not have enough resolution. We have to group the isomers in families and identify the distinctive contribution of each group of families or the average spectrum for each group. Therefore, we are going to fit the experimental spectrum in a more general way. We can assume that the contribution from OH·N^9^ interaction mainly lies in the absorption spectrum between 3320–3420 cm^−1^ (blue trace in Figure 4), O^2^H·OC group contributes to the range 3380–3480 cm^−1^ (red trace in Figure 4) and O^6^H·OC group contributes from 3450 up to 3520 cm^−1^ (grey trace in Figure 4). This experimental and computational comparison mainly points to the presence of the blue (intramolecular stabilisation) and red (intermolecular stabilisation) families. The computational spectra of each isomer (Figures S2–S23), the average spectra of each family (13 families reduced to 12 because of similarities, Figure S29) and the average spectra by group of families (5 groups of families, Figure S30) at the two theory levels are reported in the Supplementary Materials. Even if we computed all these calculations, we prefer to prevent the over interpretations and we only assume a general inclusive assignment for the conclusions. In summary, regarding the precision of the prediction, it is remarkable to note that not only the contribution of the global minimum structure (OH·N^9^ family) is needed to explain the experimental spectrum but also that of the two families with the strongest intermolecular interaction (O^2^HOC and O^6^HOC) are mandatory. Therefore, the interactions required to simulate the spectrum are precisely those found in the most stable structure and in the two species with the strongest intermolecular interaction.

**Figure 5:**
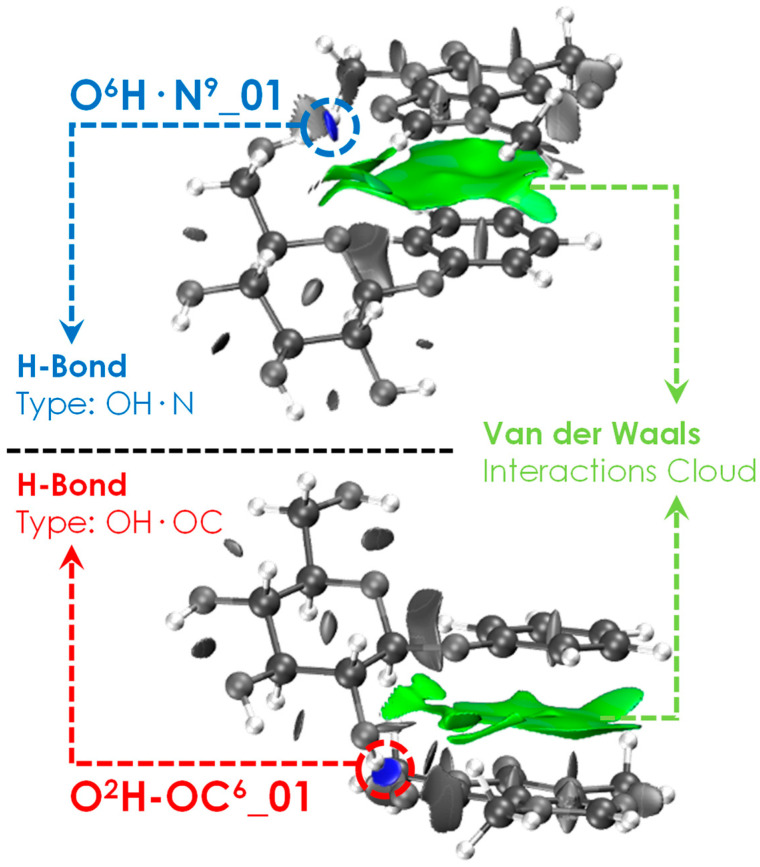
NCI plots (iso value *s* = 0.5) of the most stable (RE: O^6^H·N^9^_01) and that of the most strongly bonded (BE: O^2^H·OC^6^_01) structures. The original pictures together with the 2D-graphics are reported in Figure S31.

The assignment of the experimental spectrum also reveals important information about the stacking of Caf with the aromatic ring of PhGlc. All stacked orientations adopted by Caf are perfectly stable, regardless of its orientation with respect to the π-cloud. Caf floats above the electronic cloud, and it is blocked in a fixed conformation only when one of its proton-acceptor groups interacts with a hydroxyl group of glucose. This interpretation is reinforced by the assignment of all three interaction types (O^6^H·OC, O^2^H·OC and OH·N^9^), regardless of the interaction side of Caf with the aromatic ring of PhGlc. These interactions have been highlighted in the NCI images (see Figure 5), in which we can observe in green the stacking interactions and in blue the hydrogen bonds characterising the most stable conformations O^6^H·N^9^_01 and O^2^H·OC^6^_01. Additional figures and graphics about the NCI analysis are available in Figure S31.

## 3. Discussion

### 3.1. General Tendency in the Interaction with Caffeine

**Figure 6:**
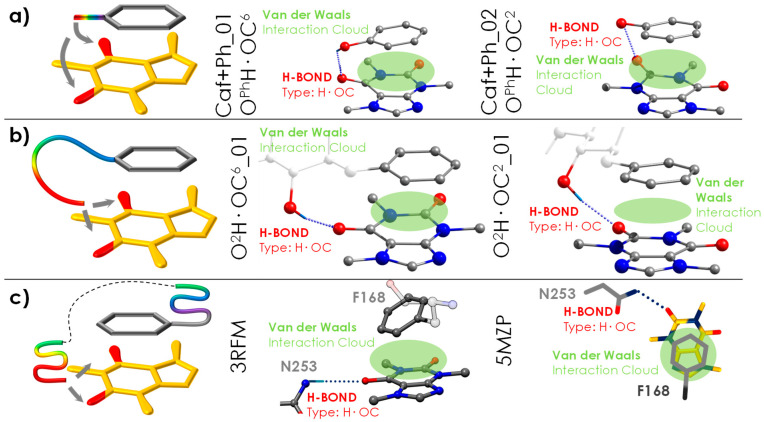
(**a**) The most strongly bonded conformations of Caf+Ph (Caf+Ph_01 and Caf+Ph_02) [15]. (**b**) The most strongly bonded conformations of Caf+PhGlc (O_2_H·OC^2^_01 and O_2_H·OC^6^_01). (**c**) The comparison of the structures is extended to the A_2A_ receptor. The PDB codes of the corresponding structures are 3RFM [8] and 5MZP [9].

In a previous publication, the molecular interactions between Caf and Ph were analysed (see Figure S29) [15]. We compare the section of common structures of Caf+Ph and Caf+PhGlc complexes looking at their root mean square deviation (RMSD) obtained by the superposition of their atoms (for more details, see Figure S30). They reveal important similarities in the π···π stacking interactions. It is possible to observe how Ph tries to produce two interactions simultaneously, hydrogen bond and π···π stacking interactions, but for the Caf+Ph complex, this is prevented by the short distance between the aromatic ring and the hydroxyl group. In the case of phenylated glucose, the molecule increases the distance between the hydroxyl and the aromatic ring, gaining flexibility; this allows the maximisation of the interaction (Figure 6). The interactions observed in the receptor described in the following section follow this same trend in order to produce both π···π stacking interaction and hydrogen bond.

### 3.2. General Tendency and the Similarities with the Interaction inside the A_2A_ Receptor

The results obtained can be used to better understand the non-selective antagonistic behaviour of Caf inside the adenosine receptor. A direct interaction between Caf and the phenyl group of the F168 residue was previously reported [8,9]. This residue acts as an anchor point for the ligands in the receptor, allowing the accommodation of hydrophobic and planar molecules but in a nonselective way [8,9]. The selectivity of the docking comes from the hydrogen bonds, which can only take place if the ligand presents proton-accepting functional groups in the correct positions. Interestingly, a similar situation is observed in the dimer analysed in this work: the stacking interaction between the two molecules is somehow unspecific and the different families differ only in the network of intermolecular hydrogen bonds. The intermolecular interactions observed in the detected species (the most bonded ones, O^2^H·OC^6^_01 and O^2^H·OC^2^_01, see Figure 6b) match similarly to those in Caf+Ph (Caf+Ph_01 and Caf+Ph_02, see Figure 6a) [15].

The comparison between the structure of the Caf+PhGlc dimer and the interactions of Caf inside the orthosteric site of the A_2A_ receptor (see Figure 6c) uncovers surprising similarities, mainly because the noncovalent interaction pattern is maintained. In the case of Caf·PhGlc (Figure 6b), the molecular flexibility of the partner (PhGlc) results in several families of structures, as demonstrated by the experimental spectrum. In particular, both families (O^2^H·OC^6or2^) stand out with significantly higher binding energy values, approximately 10 kJ/mol above that of the global minimum. Surprisingly, the same two structural families are in agreement with the conformational arrangement adopted by Caf inside the orthosteric site of the A_2A_ receptor (see Figure 6c, where the PDB structures are displayed, and a more detailed comparative analysis is shown in Figure S31).

Actually, it seems that the rules determining the preference between stability and binding energy are the same as those that govern also the disposition of the ligand inside the receptor. In the presence of water and at room temperature, the determinant factor in the docking process is how strongly the ligand can bind to the receptor, because such binding has to compensate for the desolvation of both ligand and receptor.

## 4. Materials and Methods

### 4.1. Computations

Detailed description of the computational protocol may be found in previous publications [15,28]. The specific procedures used for the studied systems are widely detailed in the Supplementary Materials in the computational Methods section. First, all possible conformations within a given energy window were located in a conformational search, carried out using MMFFs [29], OPLS3e [30] and AMBER [31] force fields, as implemented in Macromodel (Schrödinger Suite) [32]. Then, we subtracted the redundant structures by a clustering process. The preselected candidates were further optimised and the frequency calculated at the M06-2X/6-311++G(d,p) and B3LYP-ED=GD3BJ/def2TZVP levels. In a second screening procedure, structural candidates that evolved to the same optimised geometries were subtracted. Once all the minima were obtained, the main variable for the classification of the structures in families is identified, which corresponds to how the intermolecular interaction is established inside the complex.

The optimised structures were carefully analysed in order to build the iPES diagram of the complex, where each structure was labelled and classified depending on its interaction type. To build the iPES, we connect in a diagram the intermolecular families with the closest structural family (see Figure 2). Relative energies and binding energies were computed, including the ZPE and BSSE via counterpoise correction method. We analysed the stability and binding energy for each conformation for all temperature ranges, from 0 to 700 K, which is the temperature at which approximately the organic matter decomposes. The temperature effects were included by using the rigid rotor and harmonic oscillator approximation, so some deviations at high temperature can occur. Despite this, the qualitative trend of results remains reliable. All the information is available in the Supplementary Material: the families are in Table S1, and the conformation information is in Figures S1–S23. Furthermore, the simulation of the theoretical spectra is also reported in the Supplementary Materials. These simulations for the IR spectra were performed applying an experimentally determined scaling factor [15] to the results obtained from the normal mode analysis using the Gaussian16TM software package (G16, Rev. C.01) [33]. The bands were simulated using Lorentzian functions (FMHW of 5 cm^−1^ for free NH/OH groups and a wider width for modes involved in an interaction), convolved with a Gaussian function (FMHW = 6 cm^−1^) in order to simulate the broadening effect of the laser. We also averaged the theoretical spectrum for each family. To give an even more general view, we grouped families by similar interaction functional groups to create groups of families, and the average spectrum of these groups of families is also depicted. For the binding energy calculation, we relied on the complexation reaction between Caf and PhGlc (PhGlc is in the most similar stable conformation compared with the arrangement in the complex): they react with each other to form the complex. A more detailed explanation about the binding energy can be found in Figure S32 of the Supplementary Materials.

### 4.2. Experimental Setup

The experimental system consists of a supersonic expansion chamber coupled to a time of flight mass spectrometer (Wiley-McLaren, University of Oxford) with an in-house built laser desorption system [34]. Commercially available samples of caffeine (Sigma Aldrich, St. Louis, MO, USA, 99%) and phenyl-β-D-glucopyranoside (Sigma Aldrich, 99%), in a homogeneous mixture with carbon nanotubes (MWCNT, Cheaptubes Inc., Cambridgeport, VT, USA), used as matrix, were deposited in a cylindrical sample holder close to the nozzle exit of a pulsed supersonic valve (General Valve Series 9). Radiation from the first harmonic of an Nd/YAG laser (1064 nm, Quantel Brilliant-b) was used to ablate the sample synchronously with the valve opening, so that the supersonic expansion (created with Ar at a backing pressure of 14 bar) could pick up the vaporised material. The valve opening and supersonic expansion conditions were optimised to achieve the best conditions for the formation of Caf+PhGlc dimers. Before entering the ionisation region of the time-of-flight spectrometer, the molecular beam was collimated using a 2 mm skimmer (Beam Dynamics). Subsequently, the beam was interrogated using 1-color two-photon resonance enhanced multiphoton ionisation (1c-R2PI), with the aid of a tuneable UV laser (Fine Adjustment pumped with Quantel Brilliant-b, Coumarine 540 A, 10 Hz), in order to obtain the electronic spectrum of the neutral molecules with vibrational resolution. Structural information of the molecular systems was obtained using the resonant ion dip infrared spectroscopy technique (RIDIRS) based on IR/UV double resonance experiments [20,35]. An OPO/A laser (LaserVision, 5 Hz repetition rate) was used to generate IR radiation, which interrogated the molecular beam ~100 ns prior to the UV laser. The delays between all pulses were fine-tuned (Standford Delay Generators) to maximise the signal of the cluster of interest and to maximise the percentage of the UV signal depleted by the IR laser. Special care was taken to achieve a perfect overlap between both lasers.

## 5. Conclusions

We present a thorough exploration of the caffeine+phenyl-β-D-glucopyranoside dimer properties, using DFT calculations and laser spectroscopy in an isolated environment. Thanks to the in-depth characterisation performed in this work on the specific chemical contacts responsible for the most stable structural arrangements in an environment free of interfering factors, we proved that, in these types of biological systems, the intramolecular stability can be sacrificed in favour of the efficacy of the interactions. This is one of the few times that this chemical behaviour has been clearly revealed experimentally. The results presented here demonstrate the importance of considering not only the most stable structures but also the most tightly bonded ones in the extrapolation of the observations in isolated conditions to real biological systems. The relevant conclusions reached in this work encourage us to continue with the study of molecular aggregation using a powerful combination of supersonic expansion, laser spectroscopy and computational chemistry.

## Supplementary Materials

The following supporting information can be downloaded at: https://www.mdpi.com/article/10.3390/ijms24054390/s1. (References [36,37] are cited in the Supplementary Materials).

## References

[1] Astorino T.A., Alkadhi K.A., Addicott M., Farah A., Balssa F., Keast R.S.J., Chapman R.F., Acquas E., Reis J.P., Sen D.J., Preedy V.R. (2012). Caffeine: Chemistry, Analysis, Function and Effects.

[2] Latosińska J.N., Latosińska M., Olejniczak G.A., Seliger J., Žagar V. (2014). Topology of the Interactions Pattern in Pharmaceutically Relevant Polymorphs of Methylxanthines (Caffeine, Theobromine, and Theophiline): Combined Experimental (1H-14N Nuclear Quadrupole Double Resonance) and Computational (DFT and Hirshfeld-BASED) Study. J. Chem. Inf. Model..

[3] Nehlig A., Daval J.L., Debry G. (1992). Caffeine and the Central Nervous System: Mechanisms of Action, Biochemical, Metabolic and Psychostimulant Effects. Brain Res. Rev..

[4] Fredholm B.B., IJzerman A.P., Jacobson K.A., Linden J., Müller C.E. (2011). International Union of Basic and Clinical Pharmacology. LXXXI. Nomenclature and Classification of Adenosine Receptors—An Update. Pharmacol. Rev..

[5] Snyder S.H., Katims J.J., Annau Z., Bruns R.F., Daly J.W. (1981). Adenosine Receptors and Behavioral Actions of Methylxanthines. Proc. Natl. Acad. Sci. USA.

[6] Ribeiro J.A., Sebastiao A.M. (2010). Caffeine and Adenosine. J. Alzheimer’s Dis..

[7] McLellan T.M., Caldwell J.A., Lieberman H.R. (2016). A Review of Caffeine’s Effects on Cognitive, Physical and Occupational Performance. Neurosci. Biobehav. Rev..

[8] Doré A.S., Robertson N., Errey J.C., Ng I., Hollenstein K., Tehan B., Hurrell E., Bennett K., Congreve M., Magnani F. (2011). Structure of the Adenosine A 2A Receptor in Complex with ZM241385 and the Xanthines XAC and Caffeine. Structure.

[9] Cheng R.K.Y., Segala E., Robertson N., Deflorian F., Doré A.S., Errey J.C., Fiez-Vandal C., Marshall F.H., Cooke R.M. (2017). Structures of Human A1 and A2A Adenosine Receptors with Xanthines Reveal Determinants of Selectivity. Structure.

[10] Pyrkov T.V., Pyrkova D.V., Balitskaya E.D., Efremov R.G. (2009). The Role of Stacking Interactions in Complexes of Proteins with Adenine and Guanine Fragments of Ligands. Acta Nat..

[11] Kataev E.A., Shumilova T.A., Fiedler B., Anacker T., Friedrich J. (2016). Understanding Stacking Interactions between an Aromatic Ring and Nucleobases in Aqueous Solution: Experimental and Theoretical Study. J. Org. Chem..

[12] Tsuzuki S. (2012). CH/π Interactions. Annu. Rep. Prog. Chem.-Sect. C.

[13] Asensio J.L., Ardá A., Cañada F.J., Jiménez-Barbero J. (2013). Carbohydrate-Aromatic Interactions. Acc. Chem. Res..

[14] Tavagnacco L., Engström O., Schnupf U., Saboungi M.L., Himmel M., Widmalm G., Cesàro A., Brady J.W. (2012). Caffeine and Sugars Interact in Aqueous Solutions: A Simulation and NMR Study. J. Phys. Chem. B.

[15] Usabiaga I., Camiruaga A., Calabrese C., Maris A., Fernández J.A. (2019). Exploring Caffeine–Phenol Interactions by the Inseparable Duet of Experimental and Theoretical Data. Chem.-A Eur. J..

[16] De Vries M.S., Hobza P. (2007). Gas-Phase Spectroscopy of Biomolecular Building Blocks. Annu. Rev. Phys. Chem..

[17] Rijs A.M., Oomens J., Rijs A.M., Oomens J. (2015). IR Spectroscopic Techniques to Study Isolated Biomolecules. Gas-Phase IR Spectroscopy and Structure of Biological Molecules.

[18] Kim D., Kim H.M., Yang K.Y., Kim S.K., Kim N.J. (2008). Molecular Beam Resonant Two-Photon Ionization Study of Caffeine and Its Hydrated Clusters. J. Chem. Phys..

[19] Zhao Y., Truhlar D.G. (2008). The M06 Suite of Density Functionals for Main Group Thermochemistry, Thermochemical Kinetics, Noncovalent Interactions, Excited States, and Transition Elements: Two New Functionals and Systematic Testing of Four M06-Class Functionals and 12 Other Function. Theor. Chem. Acc..

[20] Usabiaga I., González J., Arnáiz P.F., León I., Cocinero E.J., Fernández J.A. (2016). Modeling the Tyrosine–Sugar Interactions in Supersonic Expansions: Glucopyranose–Phenol Clusters. Phys. Chem. Chem. Phys..

[21] Usabiaga I., Camiruaga A., Insausti A., Çarçabal P., Cocinero E.J., León I., Fernández J.A. (2018). Phenyl-β-D-Glucopyranoside and Phenyl-β-D-Galactopyranoside Dimers: Small Structural Differences but Very Different Interactions. Front. Phys..

[22] Su Z., Cocinero E.J., Stanca-Kaposta E.C., Davis B.G., Simons J.P. (2009). Carbohydrate-Aromatic Interactions: A Computational and IR Spectroscopic Investigation of the Complex, Methyl α-l-Fucopyranoside · Toluene, Isolated in the Gas Phase. Chem. Phys. Lett..

[23] Camiruaga A., Usabiaga I., Insausti A., León I., Fernández J.A. (2017). Sugar-Peptidic Bond Interactions: Spectroscopic Characterization of a Model System. Phys. Chem. Chem. Phys..

[24] Forsting T., Gottschalk H.C., Hartwig B., Mons M., Suhm M.A. (2017). Correcting the Record: The Dimers and Trimers of Trans-N-Methylacetamide. Phys. Chem. Chem. Phys..

[25] Hartwig B., Lange M., Poblotzki A., Medel R., Zehnacker A., Suhm M.A. (2020). The Reduced Cohesion of Homoconfigurational 1,2-Diols. Phys. Chem. Chem. Phys..

[26] Kollipost F., Otto K.E., Suhm M.A. (2016). A Symmetric Recognition Motif between Vicinal Diols: The Fourfold Grip in Ethylene Glycol Dimer. Angew. Chem..

[27] Uriarte I., Insausti A., Cocinero E.J., Jabri A., Kleiner I., Mouhib H., Alkorta I. (2018). Competing Dispersive Interactions: From Small Energy Differences to Large Structural Effects in Methyl Jasmonate and Zingerone. J. Phys. Chem. Lett..

[28] Usabiaga I., Camiruaga A., Calabrese C., Veloso A., D’mello V.C., Wategaonkar S., Fernández J.A. (2020). Exploration of the Theobromine-Water Dimer: Comparison with DNA Microhydration. Phys. Chem. Chem. Phys..

[29] Halgren T.A. (1996). Merck Molecular Force Field. I. Basis, Form, Scope, Parameterization, and Performance of MMFF94. J. Comput. Chem..

[30] Robertson M.J., Tirado-Rives J., Jorgensen W.L. (2015). Improved Peptide and Protein Torsional Energetics with the OPLS-AA Force Field. J. Chem. Theory Comput..

[31] Case D.A., Cheatham T.E., Darden T., Gohlke H., Luo R., Merz K.M., Onufriev A., Simmerling C., Wang B., Woods R.J. (2005). The Amber Biomolecular Simulation Programs. J. Comput. Chem..

[32] (2021). MacroModel and SchrödingerRelease v4, 2019.

[33] Frisch M.J., Trucks G.W., Schlegel H.B., Scuseria G.E., Robb M.A., Cheeseman J.R., Scalmani G., Barone V. Gaussian16 2016, Gaussian 16, Revision C.01. https://gaussian.com/citation/.

[34] León I., Usabiaga I., Millán J., Cocinero E.J., Lesarri A., Fernández J.A. (2014). Mimicking Anesthetic–Receptor Interactions in Jets: The Propofol–Isopropanol Cluster. Phys. Chem. Chem. Phys..

[35] Camiruaga A., Usabiaga I., D’Mello V.C., García G.A., Wategaonkar S., Fernández J.A. (2019). Revisiting the Spectroscopy of Xanthine Derivatives: Theobromine and Theophylline. Phys. Chem. Chem. Phys..

[36] Banks J.L., Beard H.S., Cao Y., Cho A.E., Damm W., Farid R., Felts A.K., Halgren T.A., Mainz D.T., Maple J.R. (2005). Integrated Modeling Program, Applied Chemical Theory (IMPACT). J. Comput. Chem..

[37] Case D., Ben-Shalom I., Brozell S., Cerutti D., Cheatham T.E., Cruzeiro V.W.D., Darden T.A., Duke R.E., Ghoreishi D., Gilson M.K. (2018). AMBER 2018.

