# Supplementary material for: A Competition between Relative Stability and Binding Energy in Caffeine Phenyl-Glucose Aggregates: Implications in Biological Mechanisms
Source: Int J Mol Sci. 2023 Feb 23;24(5):4390. doi: 10.3390/ijms24054390 (PMC10002916; doi:10.3390/ijms24054390)
Supplement: Supplementary file 1 [file ijms-24-04390-s001.zip › ijms-2209167-supplementary.pdf]

A competition between Relative Stability and Binding Energy in Caffeine aggregates: implications in biological mechanisms

Camilla Calabrese [1,†], Ander Camiruaga [2,†], Maider Parra [3], Luca Evangelisti [4], Sonia Melandri [5], Assimo Maris [5], Imanol Usabiaga [3,*] and Jose A. Fernandez [3,*]

SUPPORTING INFORMATION

[1] Departamento de Química Física y Química Inorgánica, Facultad de Ciencias - I.U. CINQUIMA, Universidad de Valladolid, E-47011 Valladolid, Spain

[2] Institut des Sciences Moléculaires d'Orsay, ISMO, UMR8214, Université Paris-Saclay, CNRS, Bat. 520, F-91405 Orsay cedex, France

[3] Departamento de Química Física, Facultad de Ciencias y Tecnología, Universidad del País Vasco (UPV/EHU), Barrio Sarriena s/N, E-48940 Leioa, Spain

[4] Dipartimento di Chimica "Giacomo Ciamician", Campus of Ravenna, Università di Bologna, via Sant'Alberto, 163, 48123, Ravenna, Italy

[4] Dipartimento di Chimica "Giacomo Ciamician", Università di Bologna, via Selmi, 2, 40126, Bologna, Italy

* Correspondence: imanol.usabiaga@ehu.eus; josea.fernandez@ehu.eus

† These authors contributed equally to this work

INDEX

COMPUTATIONAL METHODS 3
TABLE S1 3
FIGURE S1 4
FIGURE S2 5
FIGURE S3 6
FIGURE S4 7
FIGURE S5 8
FIGURE S6 9
FIGURE S7 10
FIGURE S8 11
FIGURE S9 12
FIGURE S10 13
FIGURE S11 14
FIGURE S12 15
FIGURE S13 16
FIGURE S14 17
FIGURE S15 18
FIGURE S16 19
FIGURE S17 20
FIGURE S18 21
FIGURE S19 22
FIGURE S20 23
FIGURE S21 24
FIGURE S22 25
FIGURE S23 26
FIGURE S24 27
FIGURE S25 28
FIGURE S26 29
FIGURE S27 30
FIGURE S28 31
FIGURE S29 32
FIGURE S30 33
FIGURE S31 34
FIGURE S32 35
REFERENCES 36

COMPUTATIONAL METHODS

1) Conformational search was carried out using three different force fields: MMFFs, OPLS 2005, AMBER.[1–3]
2) Clustering of the conformers into families to remove the redundant structures derived from the use of three different force fields.
3) Geometry optimizations and frequency calculations at M06-2X/6-31+G(d) level:
   172 optimized geometries, resulting in 129 isomers after elimination of 43 redundant conformations.
4) Geometry optimization and frequency calculation at M06-2X/6-311++G(d,p) level:
   129 isomers optimized that converged into 108 conformations.
5) Geometry optimization and frequency calculation of Phenol at M06-2X/6-311++G(d,p) level. This molecule only presents one isomer, for this reason is enough calculate the binding energy using only this computational method.
6) Geometry optimization and frequency calculation of phenyl-β-D-Glucopyranoside at M06-2X/6-311++G(d,p) level. This molecule presents several conformers and therefore, it is necessary to consider the specific molecular structure involved in each dimer.
7) Relative energy and binding energy calculation:
   Relative Energy of Conformer i = Conformer i Energy – Global minimum Energy
   Binding Energy of Conformer i = Conformer i Energy ($Caf+PhGlc_i$) – (Energy of molecule (Caf) + Energy of molecule ($PhGlc_i$))
8) Classification of the computed structures into families: 13 families were found.
9) Analysis of all the conformers in each family. For this purpose, the relative energies and binding energies at 0 and 298 K were considered. Using also the Nist application, the diagram for the whole temperature range was estimated.
10) Simulation of the IR spectra. A correction factor was applied to the theoretically predicted frequencies. This was obtained fitting the experimental spectrum of the monomers to the theoretically predicted frequencies at the same theory level used for geometry optimization of the dimers. The simulation was improved using a Lorentzian function for each frequency (5 cm^{-1} width for free NH/OH and with a bigger width for transitions corresponding to bonded groups) convolved with a 6 cm^{-1} width Gaussian function in order to simulate the broadening effect of the laser.
11) Examining the simulated IR spectra and the trends observed, the families can be grouped according to 5 types of interactions (Table S1).

12) Assignment of the experimental spectrum and comparison of the assigned conformations with those of Caf+Phenol dimer, based on the structures and the relative stability.

Table S1: Summary of the conformations for Caf+PhGlc dimer, attending to the type of interaction leading aggregation. After removing the redundant conformations, 108 conformers remained.

**Interaction Type (5)**	**Stacking (39)**	**$O^3H\cdots OC$ (7)**	**$O^6H\cdots OC$ (24)**	**$O^2H\cdots OC$ (20)**	**$OH\cdots N^9$ (18)**
**Families (13)**<br>Figure S3	**AgAc (5)**<br>Figure S4-5<br>**AgBc (8)**<br>Figure S6-7<br>**BgAc (11)**<br>Figure S8-9<br>**BgBc (15)**<br>Figure S10-11	**$O^3H\cdots OC^2$ (6)**<br>Figure S12-13<br>**$O^3H\cdots OC^6$ (1)**<br>Figure S12-13	**$O^6H\cdots OC^6$ (14)**<br>Figure S14-15<br>**$O^6H\cdots OC^2$ (10)**<br>Figure S16-17	**$O^2H\cdots OC^6$ (7)**<br>Figure S18-19<br>**$O^2H\cdots OC^2$ (13)**<br>Figure S20-21	**$O^6H\cdots N^9$ (8)**<br>Figure S22-23<br>**$O^2H\cdots N^9$ (9)**<br>Figure S24-25<br>**$O^6H\cdots N^9\_O^2H\cdots OC^6$ (1)**<br>Figure S22-23

Figure S1: Complete interaction potential energy surface (iPES) of the complex Caf+PhGlc. The isomers were classified by their interaction type, and connected to each other by isomerization pathways. Each structure is represented with its relative energy in two computational levels: +) M06-2X/6-311++G(d,p) and *) B3LYP-GD3BJ/def2tzvp. Energy values are given in kJ/mol.

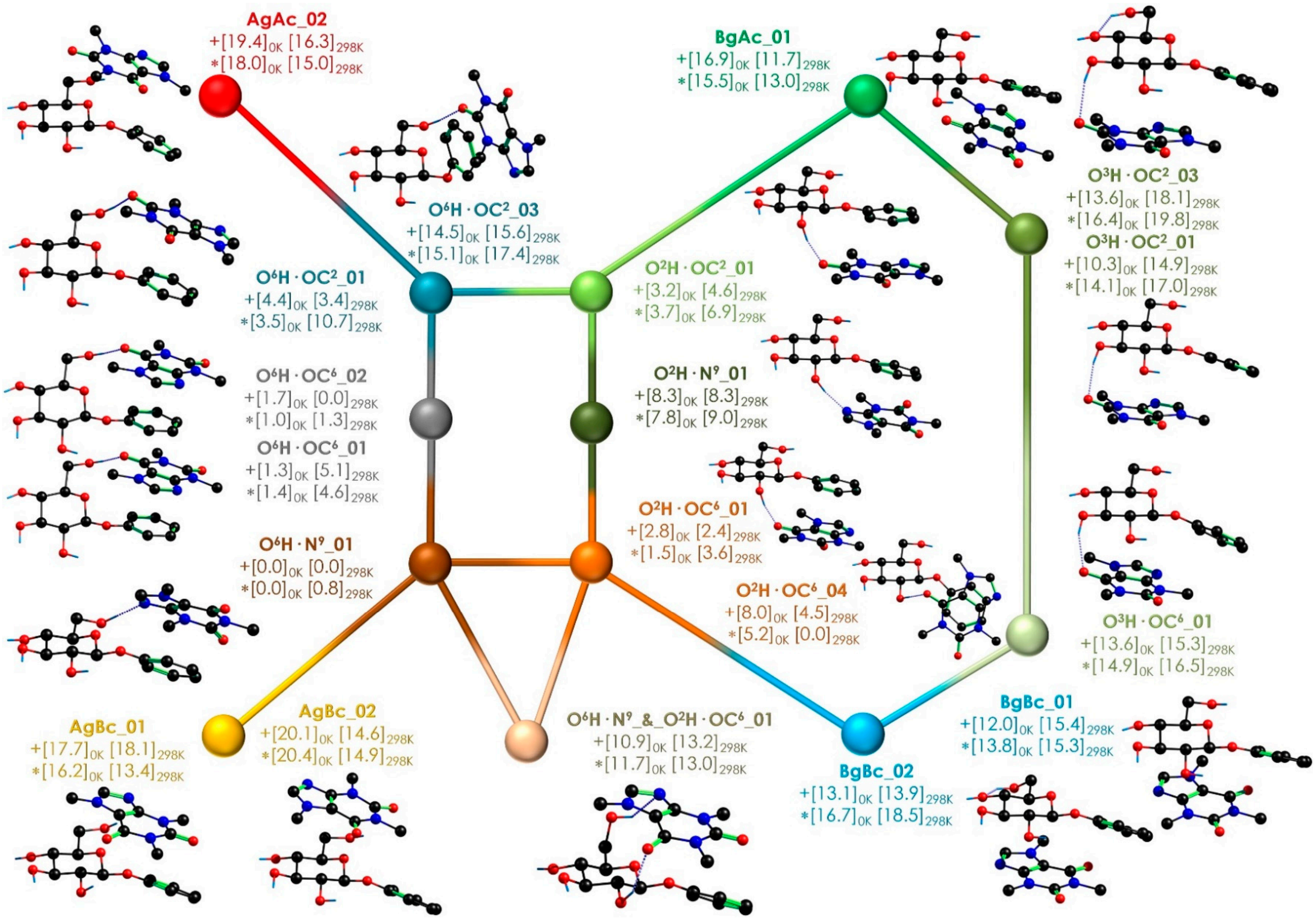

Figure S2: a) Complete set of structures of the AgAc family (face A of PhGlc (g) interacts with face A of Caf (c)), calculated at M06-2X/6-311++G(d,p) level. The first two values below each structure are the relative energy at 0 and 298 K, while the second pair of values correspond to the binding Gibbs free energy values; b) Comparison between the experimental spectrum (black trace) and the spectrum predicted for each conformation (red traces); c) Relative energy diagram calculated for the whole temperature range, referred to the global minimum; d) Relative binding Gibbs free energy diagram calculated for the whole temperature range.

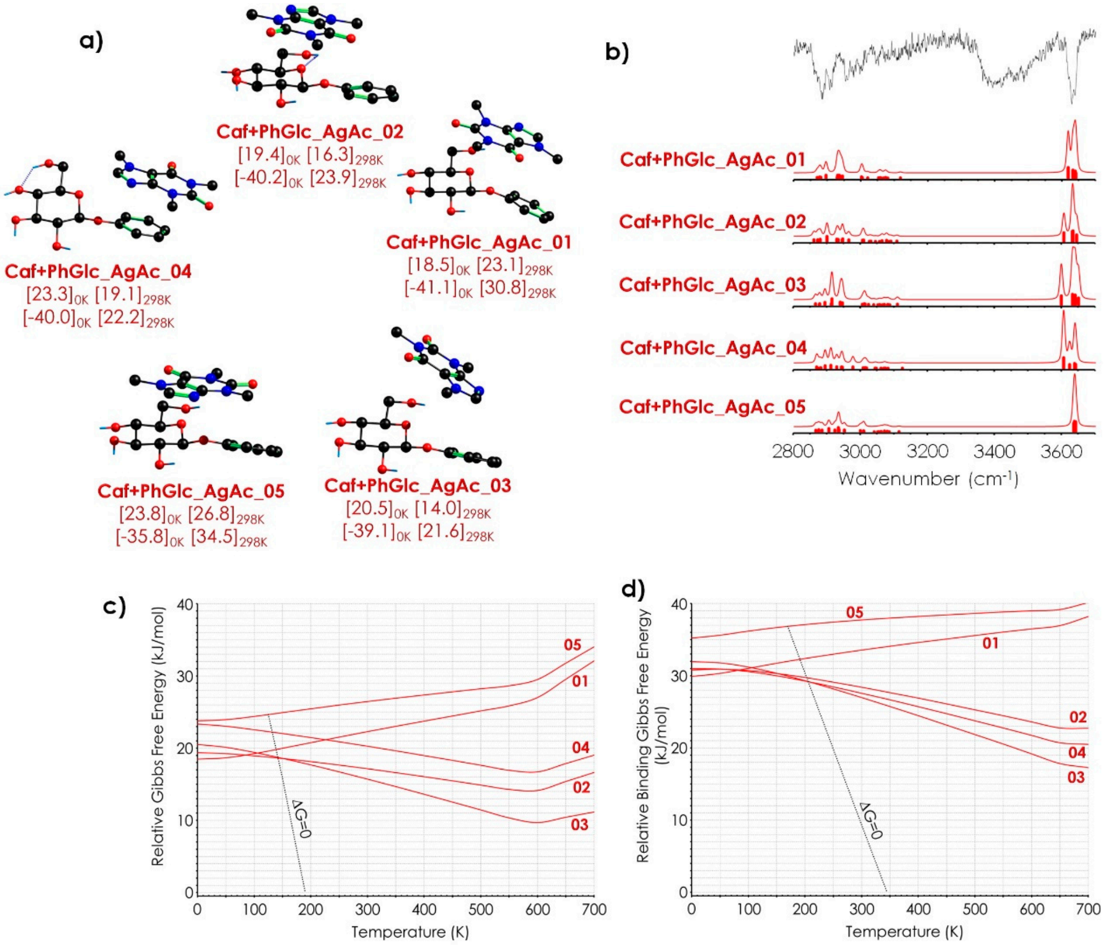

Figure S3: a) Complete set of structures of the AgAc family (face A of PhGlc (g) interacts with face A of Caf (c)), calculated at B3LYP-GD3BJ/def2TZVP level. The first two values below each structure are the relative energy at 0 and 298 K, while the second pair of values correspond to the binding Gibbs free energy values; b) Comparison between the experimental spectrum (black trace) and the spectrum predicted for each conformation (red traces); c) Relative energy diagram calculated for the whole temperature range, referred to the global minimum; d) Relative binding Gibbs free energy diagram calculated for the whole temperature range.

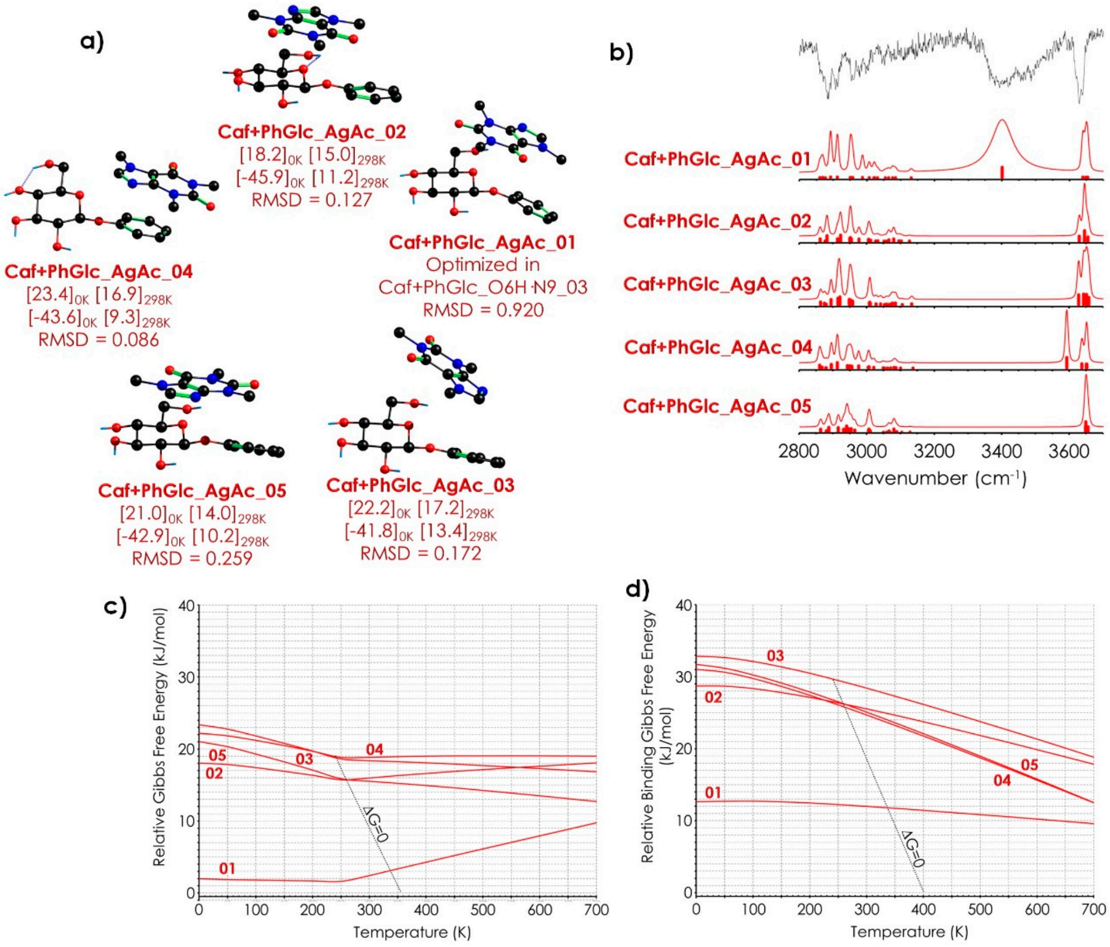

Figure S4: a) Complete set of structures of the AgBc family (face A of PhGlc (g) interacts with face B of Caf (c)), calculated at M06-2X/6-311++G(d,p) level. The first two values below each structure are the relative energy at 0 and 298 K, while the second pair of values correspond to the binding Gibbs free energy values; b) Comparison between the experimental spectrum (black trace) and the spectrum predicted for each conformation (yellow traces); c) Relative energy diagram calculated for the whole temperature range, referred to the global minimum; d) Relative binding Gibbs free energy diagram calculated for the whole temperature range.

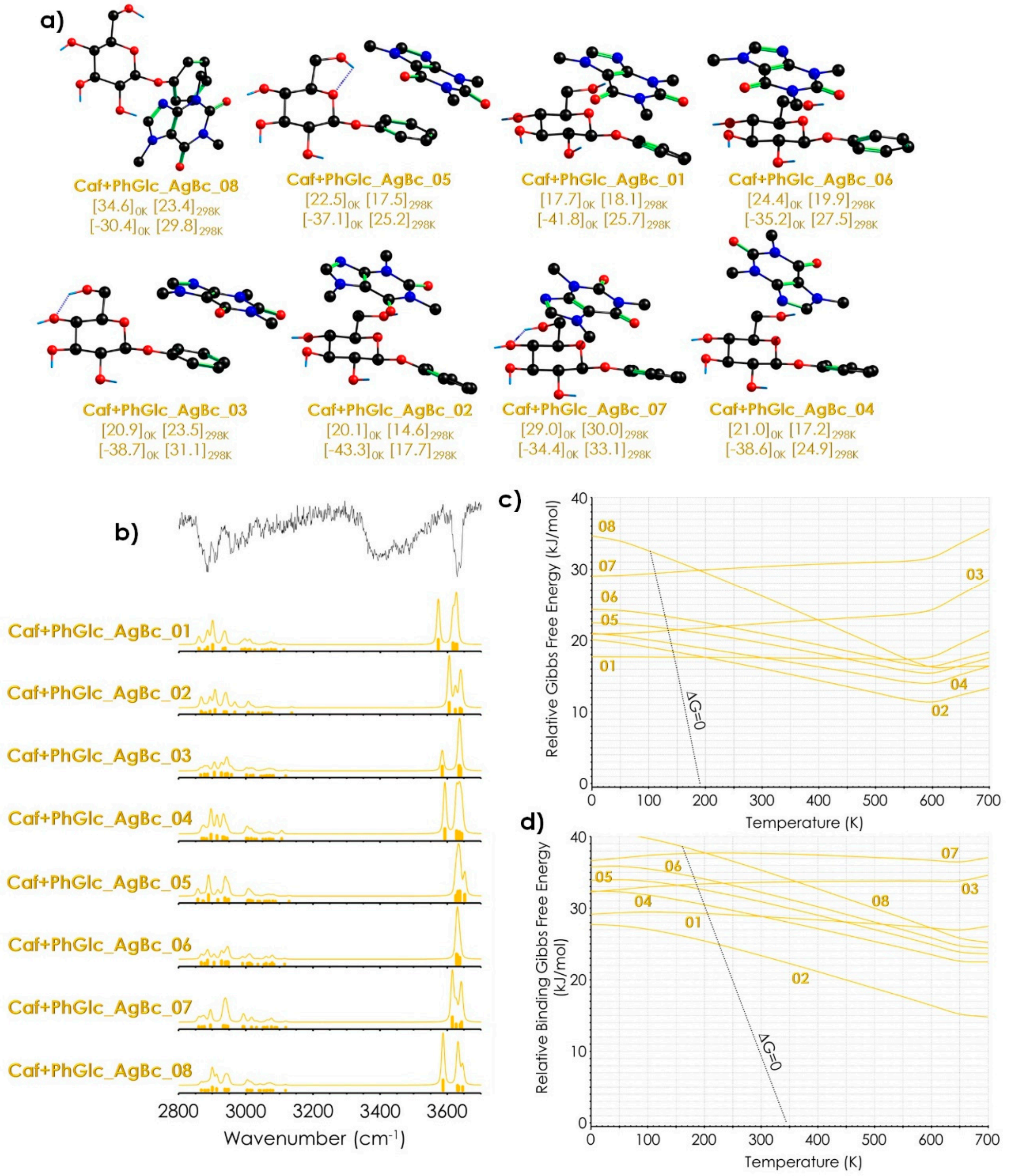

Figure S5: a) Complete set of structures of the AgBc family (face A of PhGlc (g) interacts with face B of Caf (c)), calculated at B3LYP-GD3BJ/def2TZVP level. The first two values below each structure are the relative energy at 0 and 298 K, while the second pair of values correspond to the binding Gibbs free energy values; b) Comparison between the experimental spectrum (black trace) and the spectrum predicted for each conformation (yellow traces); c) Relative energy diagram calculated for the whole temperature range, referred to the global minimum; d) Relative binding Gibbs free energy diagram calculated for the whole temperature range.

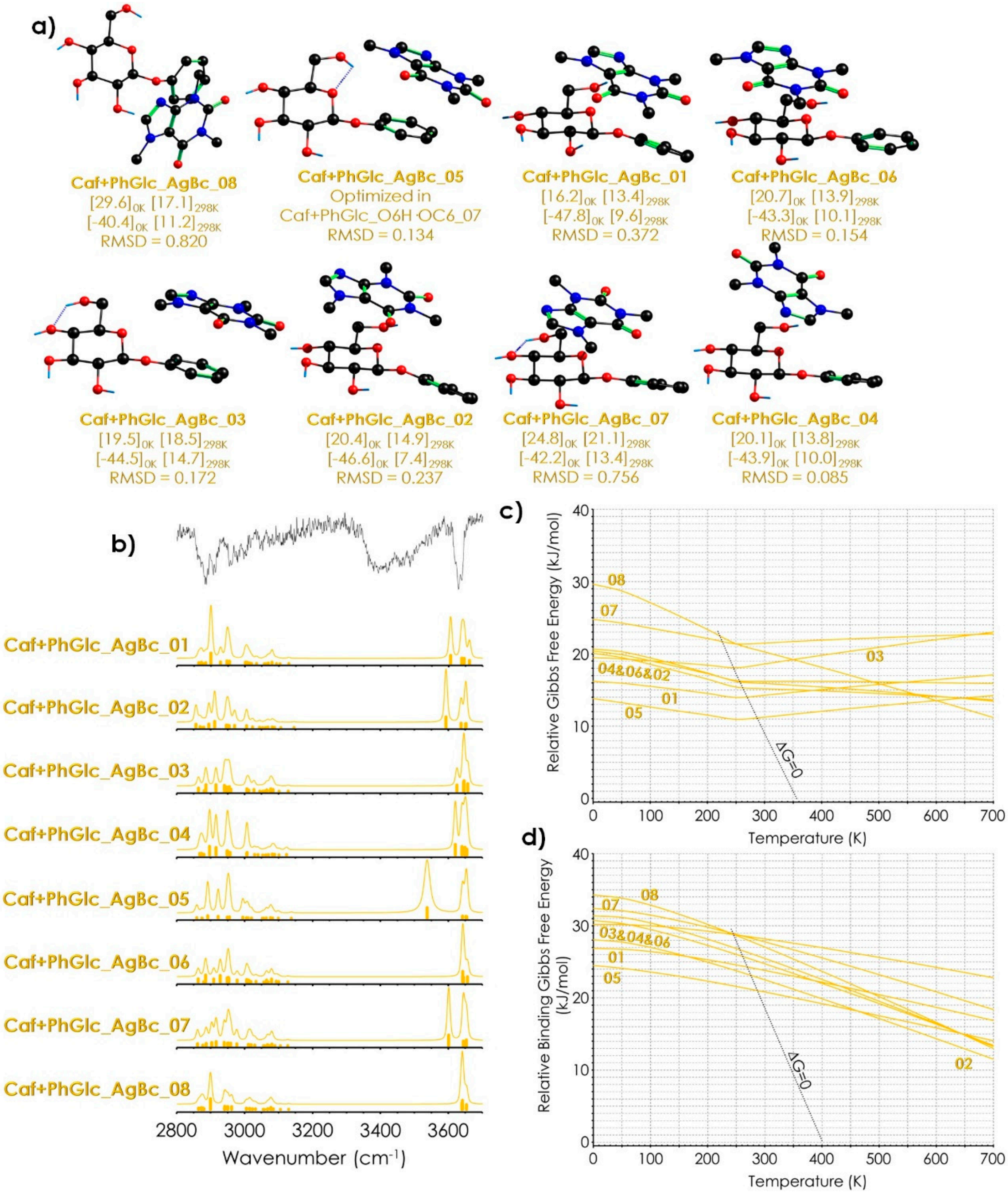

Figure S6: a) Complete set of structures of the BgAc family (face B of PhGlc (g) interacts with face A of Caf (c)), calculated at M06-2X/6-311++G(d,p) level. The first two values below each structure are the relative energy at 0 and 298 K, while the second pair of values correspond to the binding Gibbs free energy values; b) Comparison between the experimental spectrum (black trace) and the spectrum predicted for each conformation (green traces); c) Relative energy diagram calculated for the whole temperature range, referred to the global minimum; d) Relative binding Gibbs free energy diagram calculated for the whole temperature range.

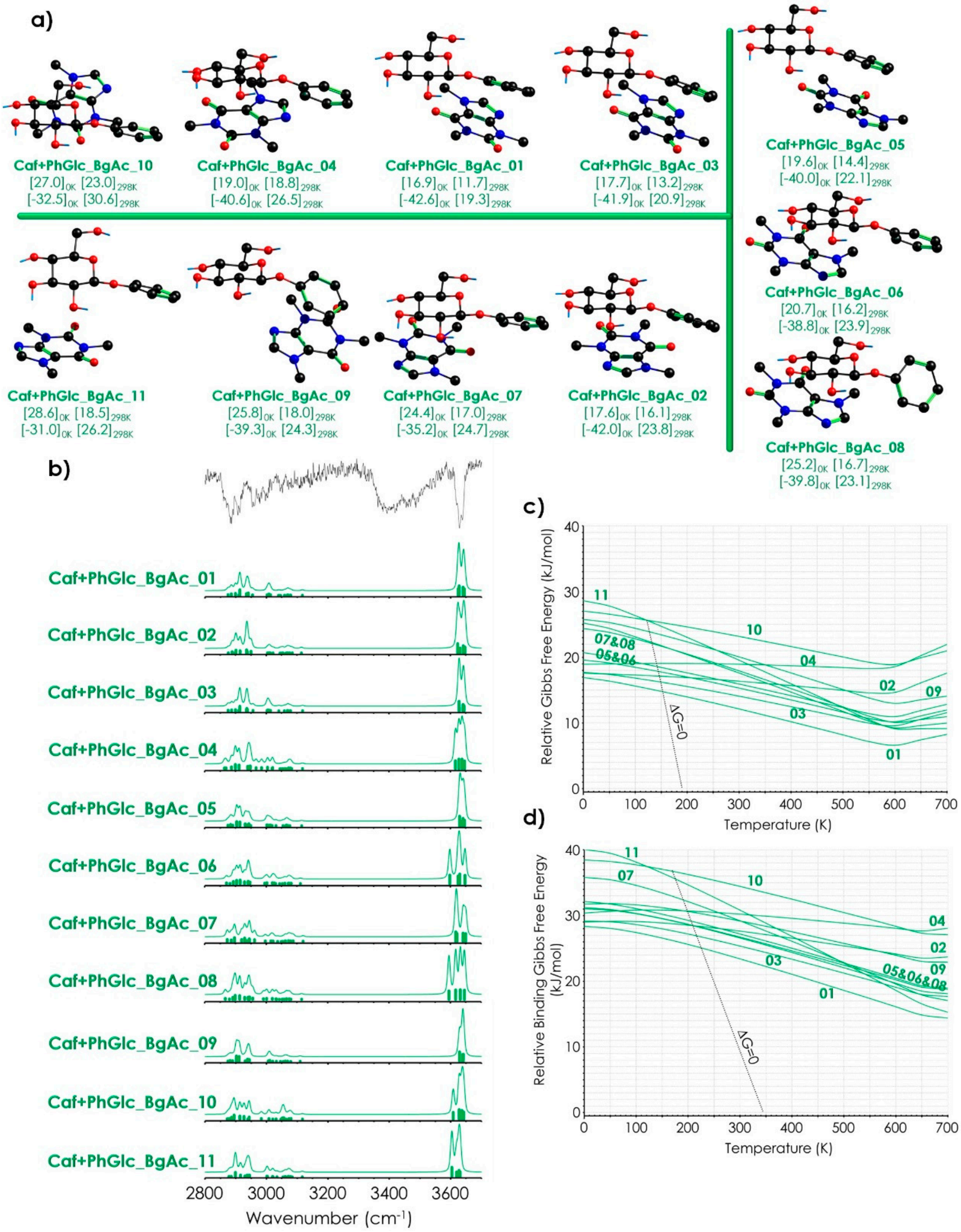

Figure S7: a) Complete set of structures of the BgAc family (face B of PhGlc (g) interacts with face A of Caf (c)), calculated at B3LYP-GD3BJ/def2TZVP level. The first two values below each structure are the relative energy at 0 and 298 K, while the second pair of values correspond to the binding Gibbs free energy values; b) Comparison between the experimental spectrum (black trace) and the spectrum predicted for each conformation (green traces); c) Relative energy diagram calculated for the whole temperature range, referred to the global minimum; d) Relative binding Gibbs free energy diagram calculated for the whole temperature range.

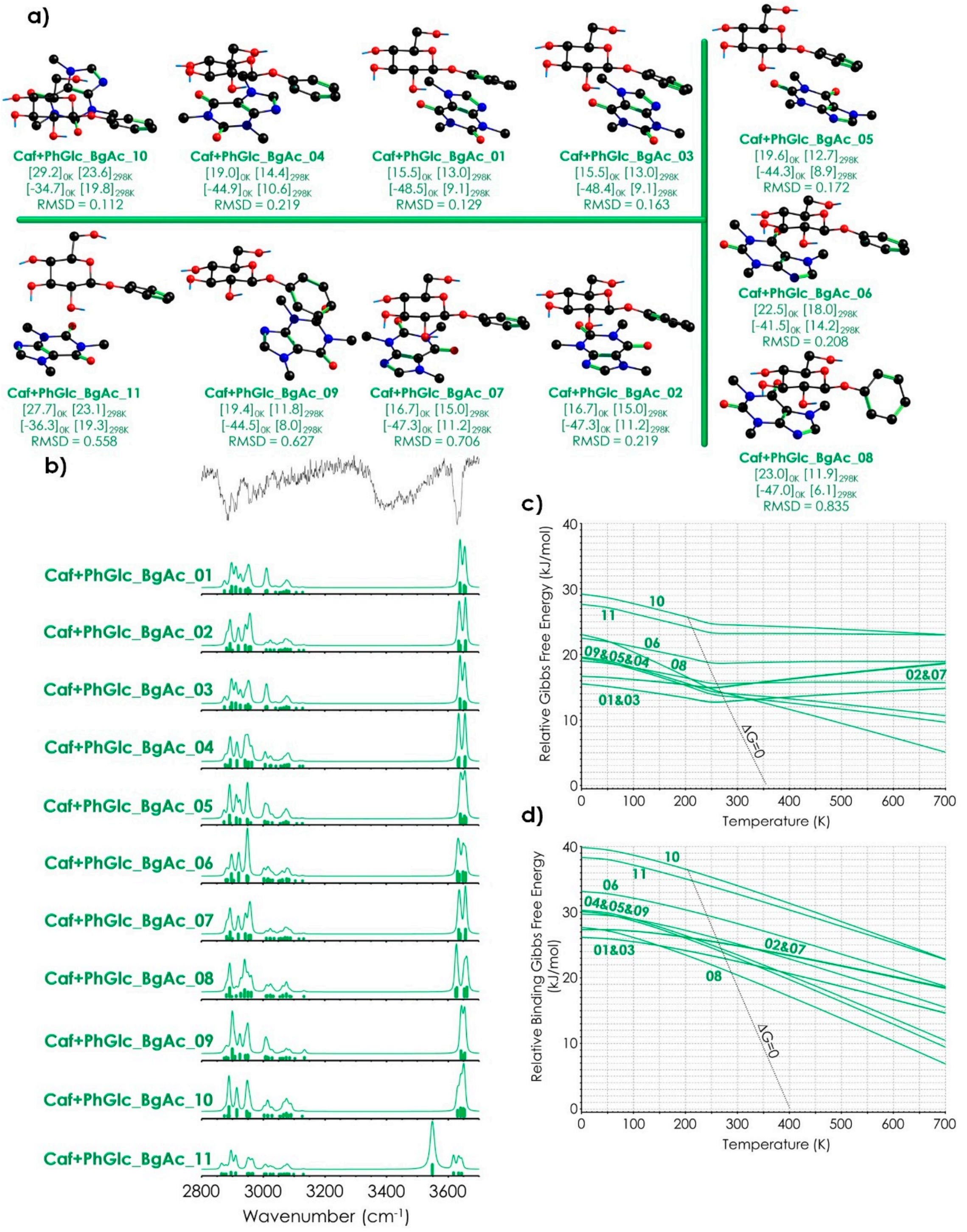

Figure S8: a) Complete set of structures of the BgBc family (face B of PhGlc (g) interacts with face B of Caf (c)), calculated at M06-2X/6-311++G(d,p) level. The first two values below each structure are the relative energy at 0 and 298 K, while the second pair of values correspond to the binding Gibbs free energy values; b) Comparison between the experimental spectrum (black trace) and the spectrum predicted for each conformation (blue traces); c) Relative energy diagram calculated for the whole temperature range, referred to the global minimum; d) Relative binding Gibbs free energy diagram calculated for the whole temperature range.

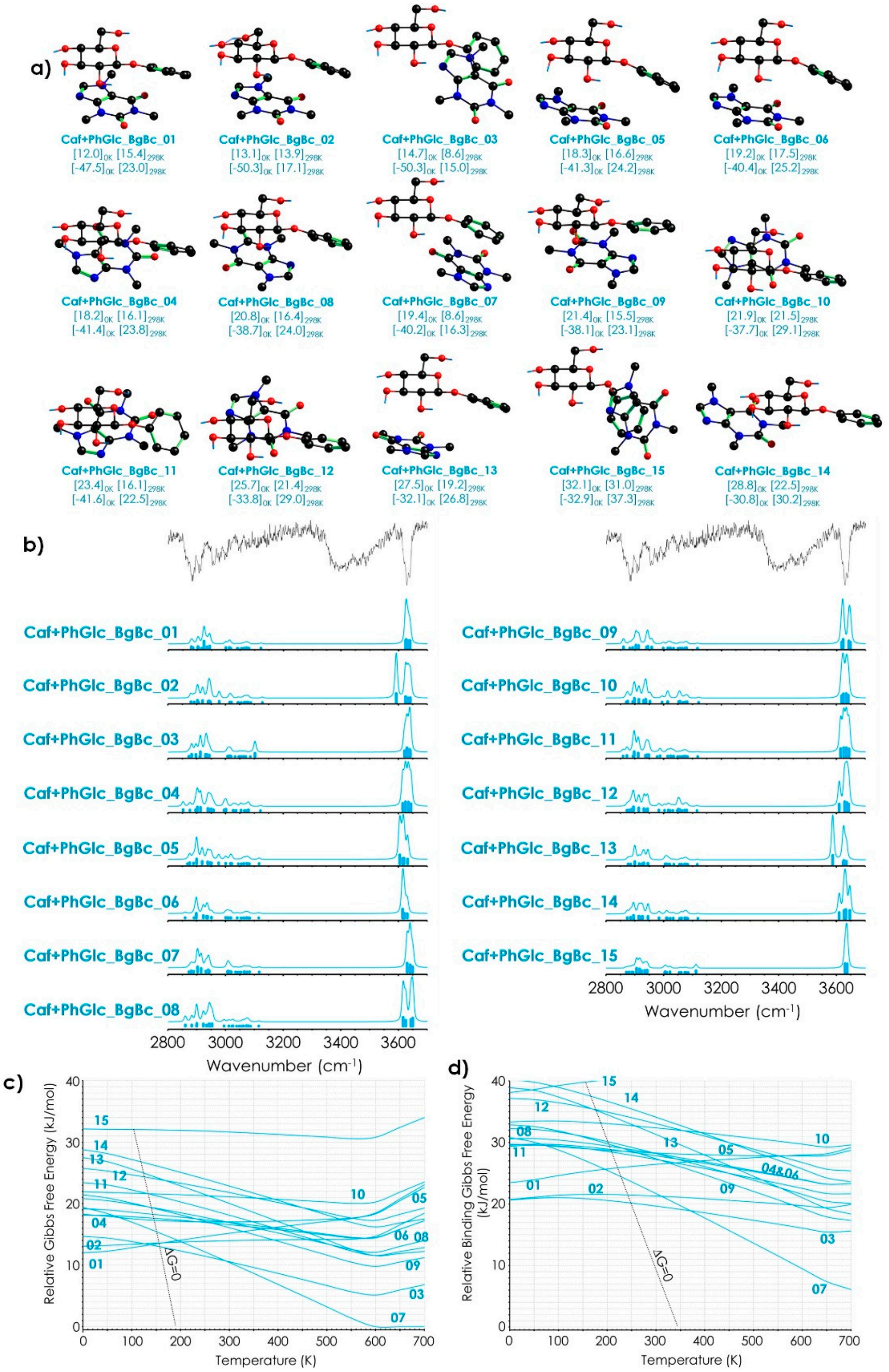

Figure S9: a) Complete set of structures of the BgBc family (face B of PhGlc (g) interacts with face B of Caf (c)), calculated at B3LYP-GD3BJ/def2TZVP level. The first two values below each structure are the relative energy at 0 and 298 K, while the second pair of values correspond to the binding Gibbs free energy values; b) Comparison between the experimental spectrum (black trace) and the spectrum predicted for each conformation (blue traces); c) Relative energy diagram calculated for the whole temperature range, referred to the global minimum; d) Relative binding Gibbs free energy diagram calculated for the whole temperature range.

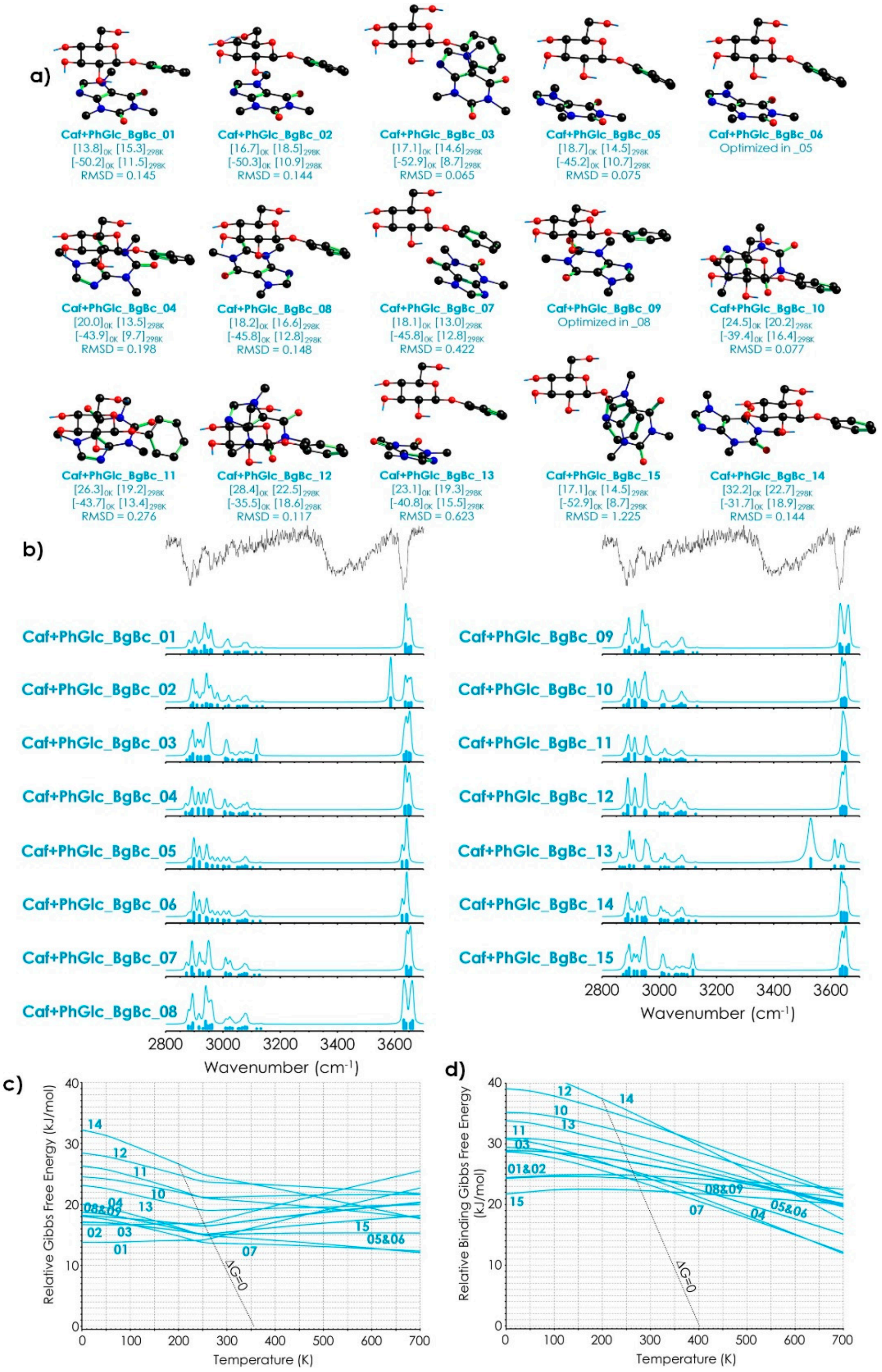

Figure S10: a) Complete set of structures of the O³H· OC² family and the single-component O³H· OC⁶ family, calculated at M06-2X/6-311++G(d,p) level. The first two values below each structure are the relative energy at 0 and 298 K, while the second pair of values correspond to the binding Gibbs free energy values; b) Comparison between the experimental spectrum (black trace) and the spectrum predicted for each conformation (green traces); c) Relative energy diagram calculated for the whole temperature range, referred to the global minimum; d) Relative binding Gibbs free energy diagram calculated for the whole temperature range.

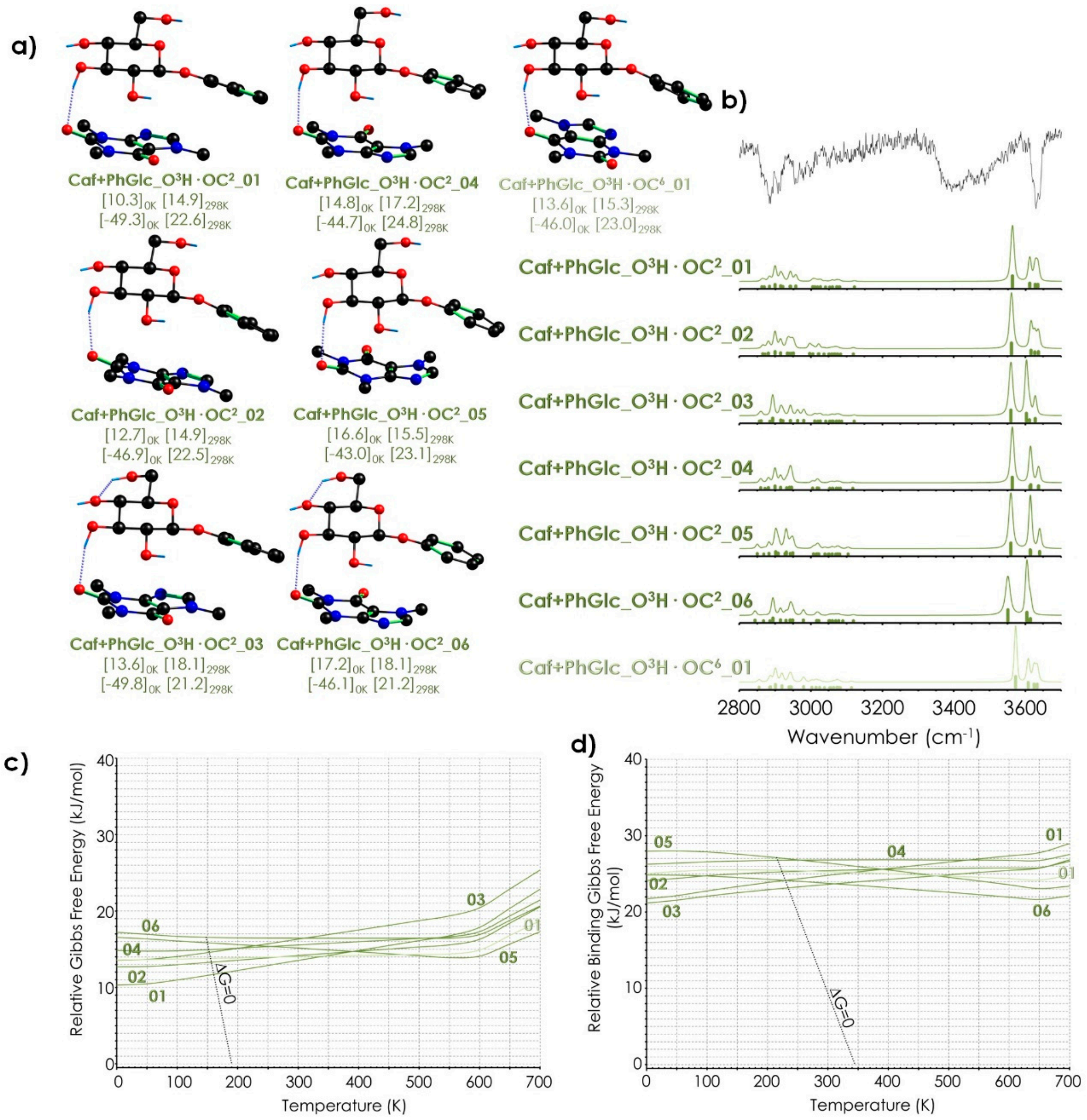

Figure S11: a) Complete set of structures of the $O^3H\cdot OC^2$ family and the single-component $O^3H\cdot OC^6$ family, calculated at B3LYP-GD3BJ/def2TZVP level. The first two values below each structure are the relative energy at 0 and 298 K, while the second pair of values correspond to the binding Gibbs free energy values; b) Comparison between the experimental spectrum (black trace) and the spectrum predicted for each conformation (green traces); c) Relative energy diagram calculated for the whole temperature range, referred to the global minimum; d) Relative binding Gibbs free energy diagram calculated for the whole temperature range.

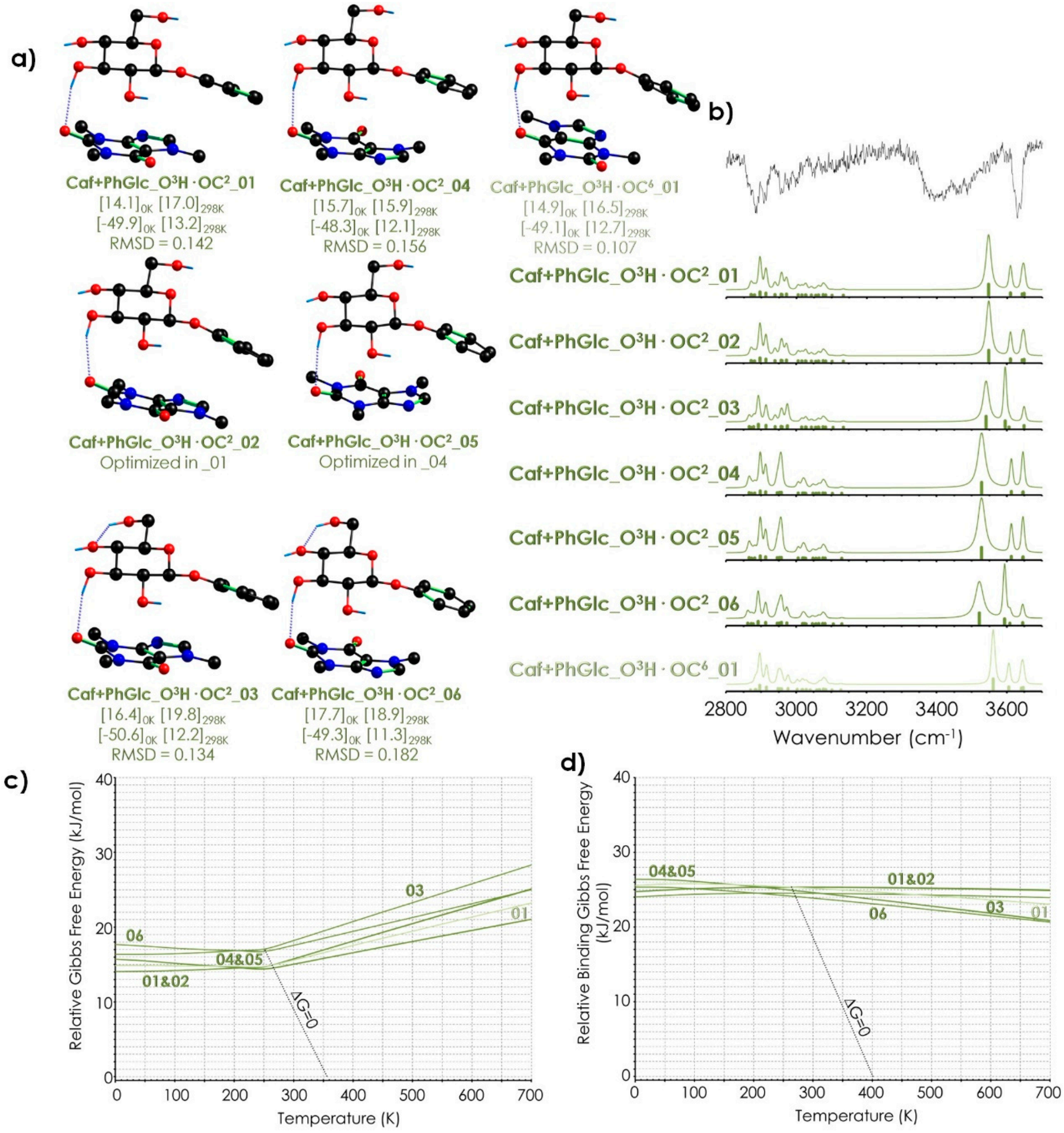

Figure S12: a) Complete set of structures of the O6H· OC6 family, calculated at M06-2X/6-311++G(d,p) level. The first two values below each structure are the relative energy at 0 and 298 K, while the second pair of values correspond to the binding Gibbs free energy values; b) Comparison between the experimental spectrum (black trace) and the spectrum predicted for each conformation (grey traces); c) Relative energy diagram calculated for the whole temperature range, referred to the global minimum; d) Relative binding Gibbs free energy diagram calculated for the whole temperature range.

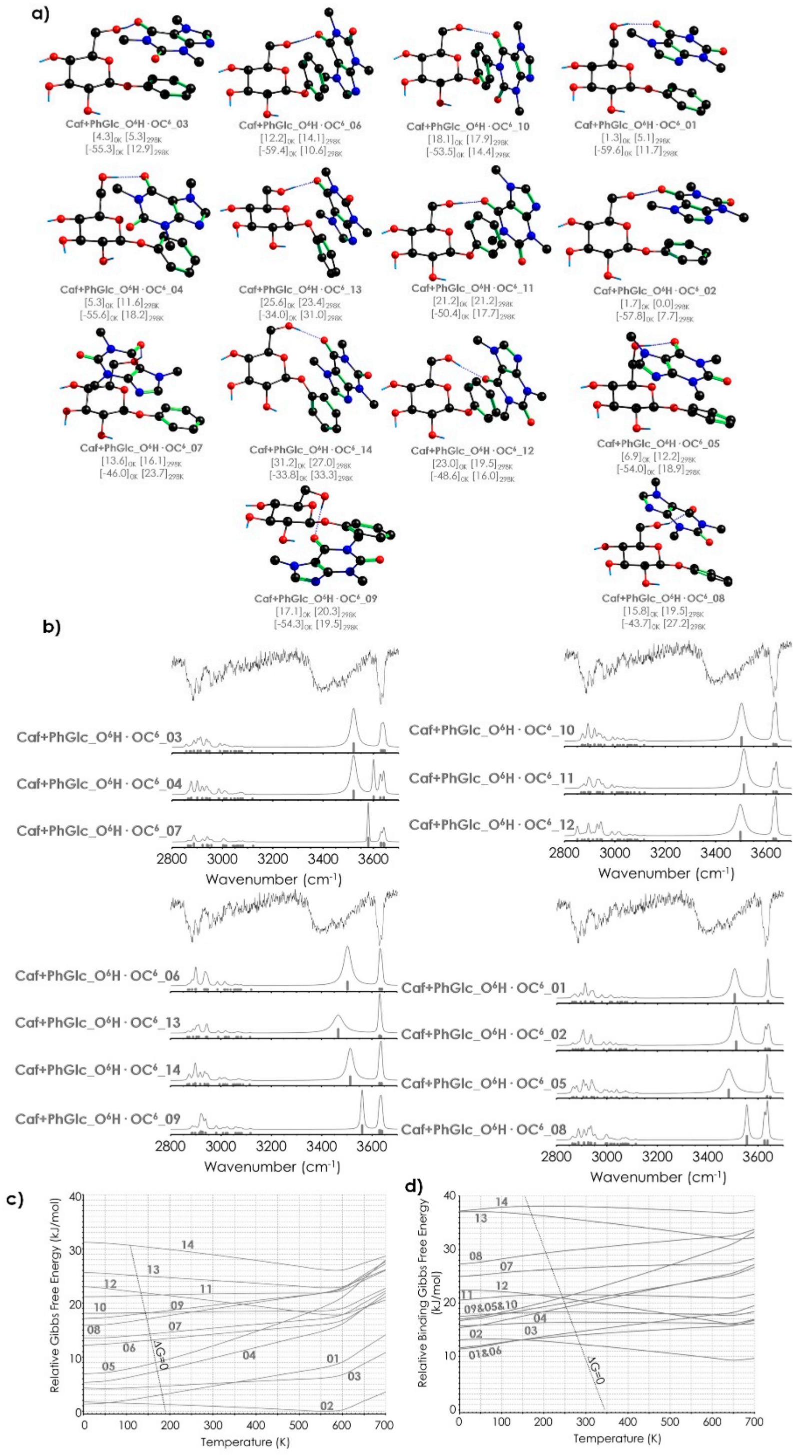

Figure S13: a) Complete set of structures of the O⁶H · OC⁶ family, calculated at B3LYP-GD3BJ/def2TZVP level. The first two values below each structure are the relative energy at 0 and 298 K, while the second pair of values correspond to the binding Gibbs free energy values; b) Comparison between the experimental spectrum (black trace) and the spectrum predicted for each conformation (grey traces); c) Relative energy diagram calculated for the whole temperature range, referred to the global minimum; d) Relative binding Gibbs free energy diagram calculated for the whole temperature range.

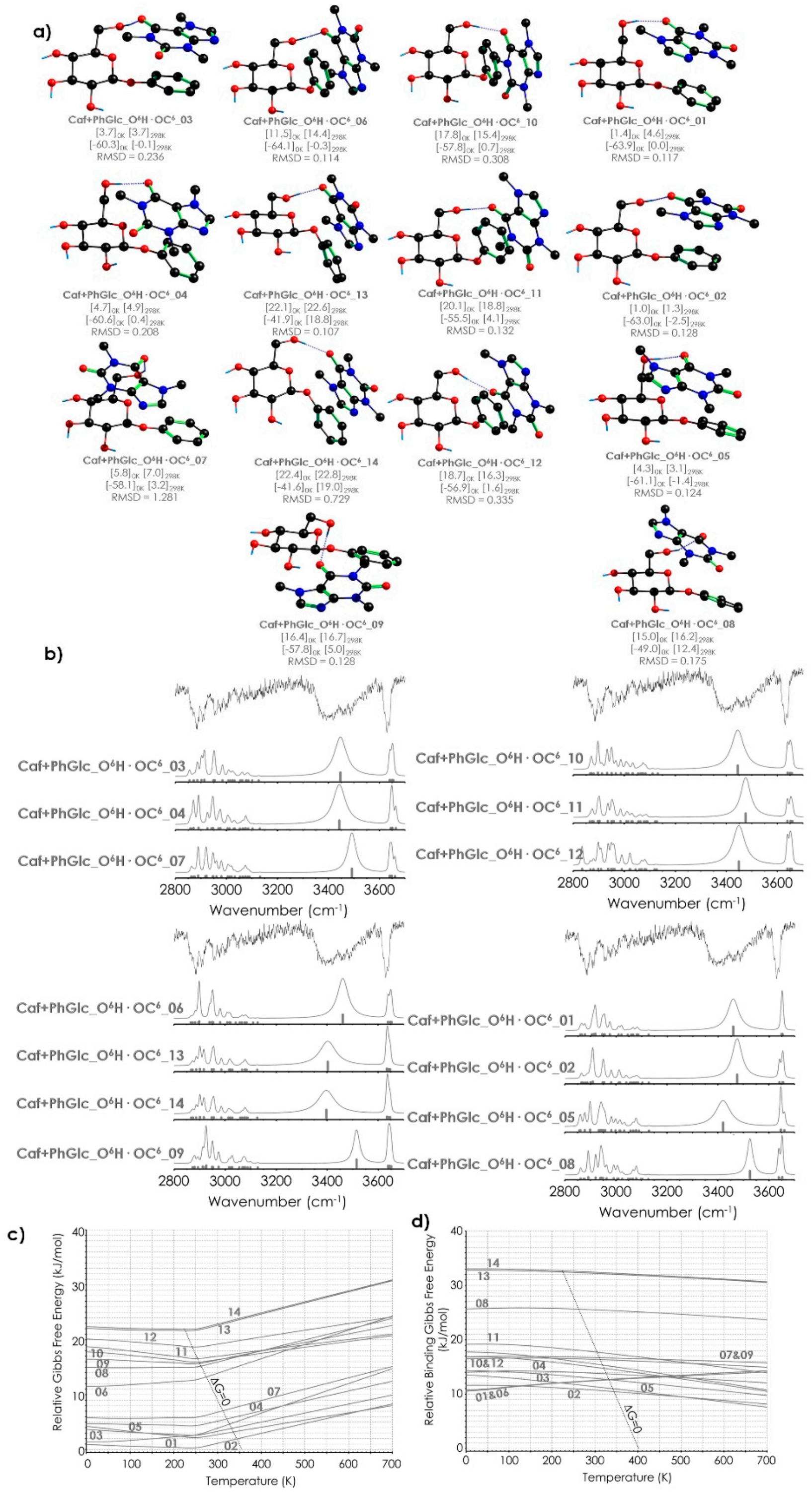

Figure S14: a) Complete set of structures of the $O^6H \cdot OC^2$ family, calculated at M06-2X/6-311++G(d,p) level. The first two values below each structure are the relative energy at 0 and 298 K, while the second pair of values correspond to the binding Gibbs free energy values; b) Comparison between the experimental spectrum (black trace) and the spectrum predicted for each conformation (blue traces); c) Relative energy diagram calculated for the whole temperature range, referred to the global minimum; d) Relative binding Gibbs free energy diagram calculated for the whole temperature range.

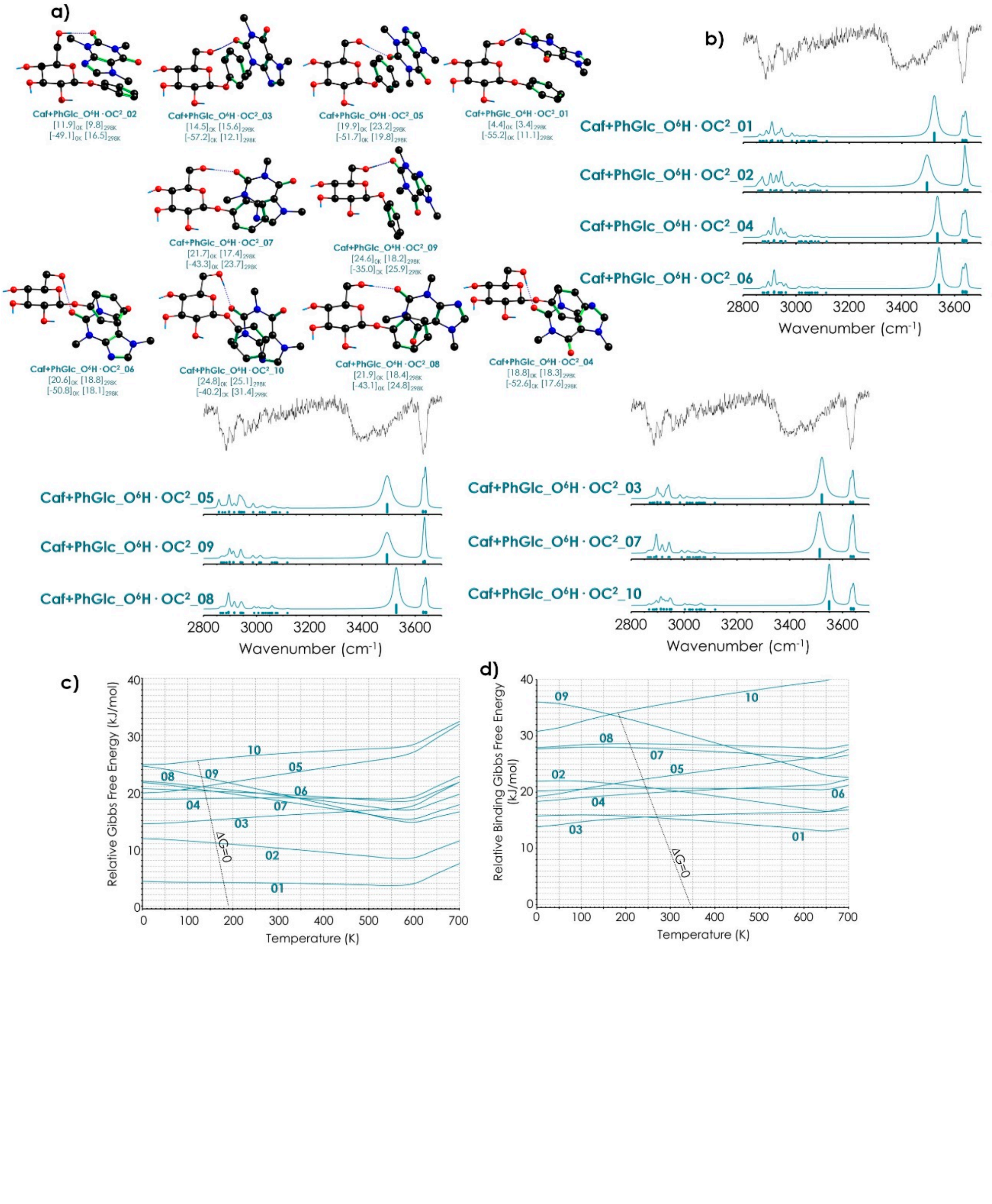

Figure S15: a) Complete set of structures of the O⁶H · OC² family, calculated at B3LYP-GD3BJ/def2TZVP level. The first two values below each structure are the relative energy at 0 and 298 K, while the second pair of values correspond to the binding Gibbs free energy values; b) Comparison between the experimental spectrum (black trace) and the spectrum predicted for each conformation (blue traces); c) Relative energy diagram calculated for the whole temperature range, referred to the global minimum; d) Relative binding Gibbs free energy diagram calculated for the whole temperature range.

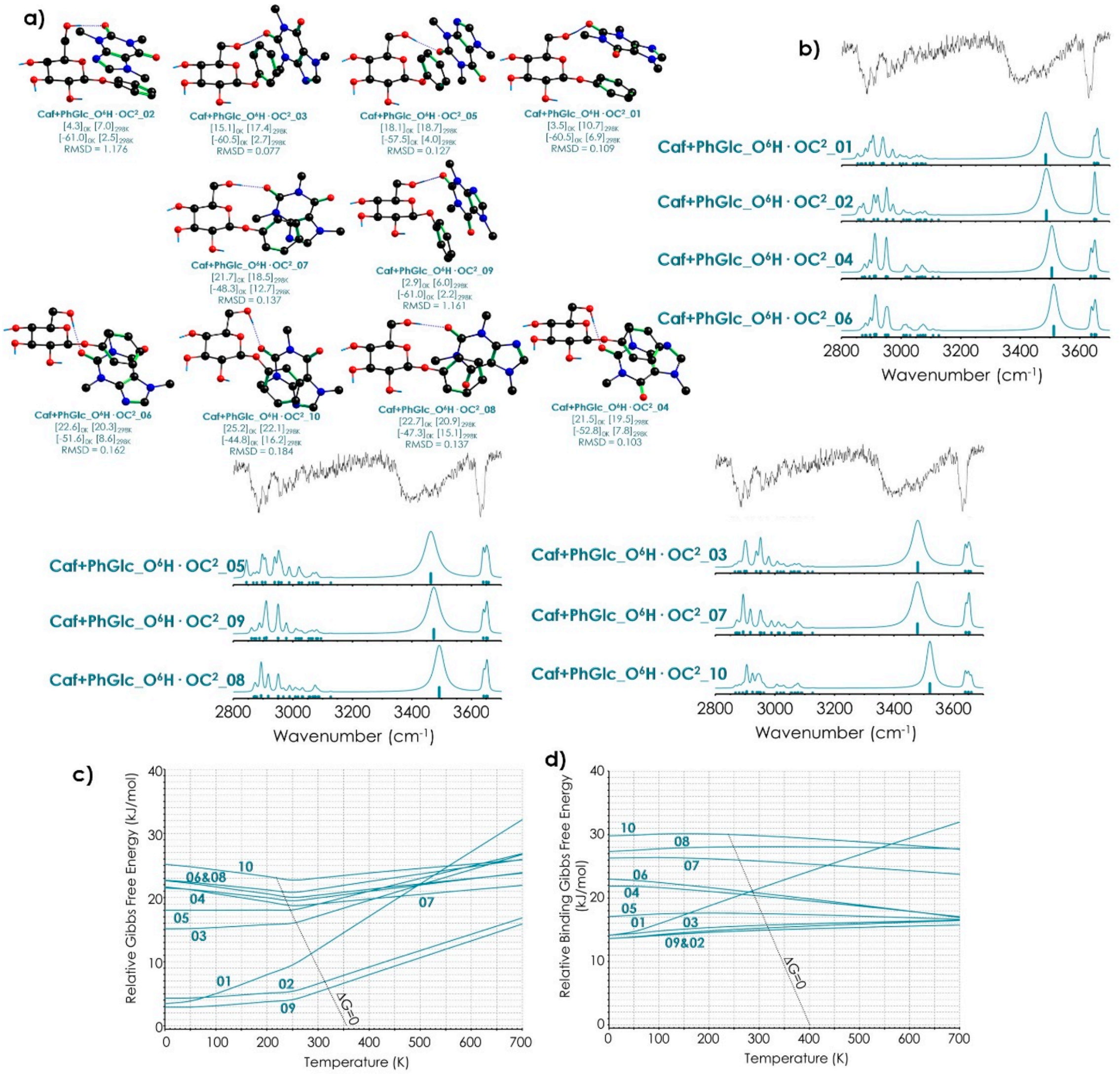

Figure S16: a) Complete set of structures of the O2H · OC6 family, calculated at M06-2X/6-311++G(d,p) level. The first two values below each structure are the relative energy at 0 and 298 K, while the second pair of values correspond to the binding Gibbs free energy values; b) Comparison between the experimental spectrum (black trace) and the spectrum predicted for each conformation (orange traces); c) Relative energy diagram calculated for the whole temperature range, referred to the global minimum; d) Relative binding Gibbs free energy diagram calculated for the whole temperature range.

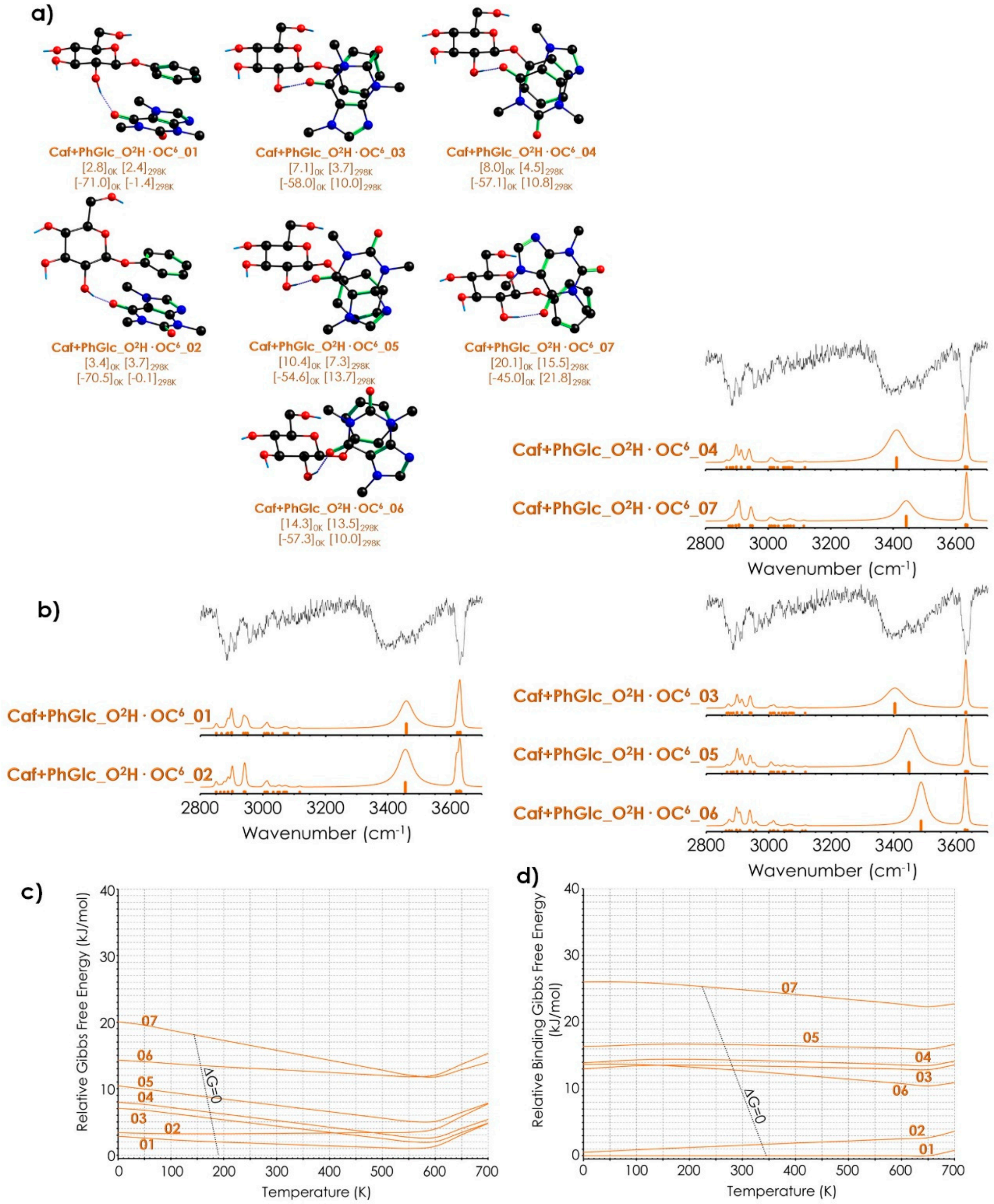

Figure S17: a) Complete set of structures of the O²H · OC⁶ family, calculated at B3LYP-GD3BJ/def2TZVP level. The first two values below each structure are the relative energy at 0 and 298 K, while the second pair of values correspond to the binding Gibbs free energy values; b) Comparison between the experimental spectrum (black trace) and the spectrum predicted for each conformation (orange traces); c) Relative energy diagram calculated for the whole temperature range, referred to the global minimum; d) Relative binding Gibbs free energy diagram calculated for the whole temperature range.

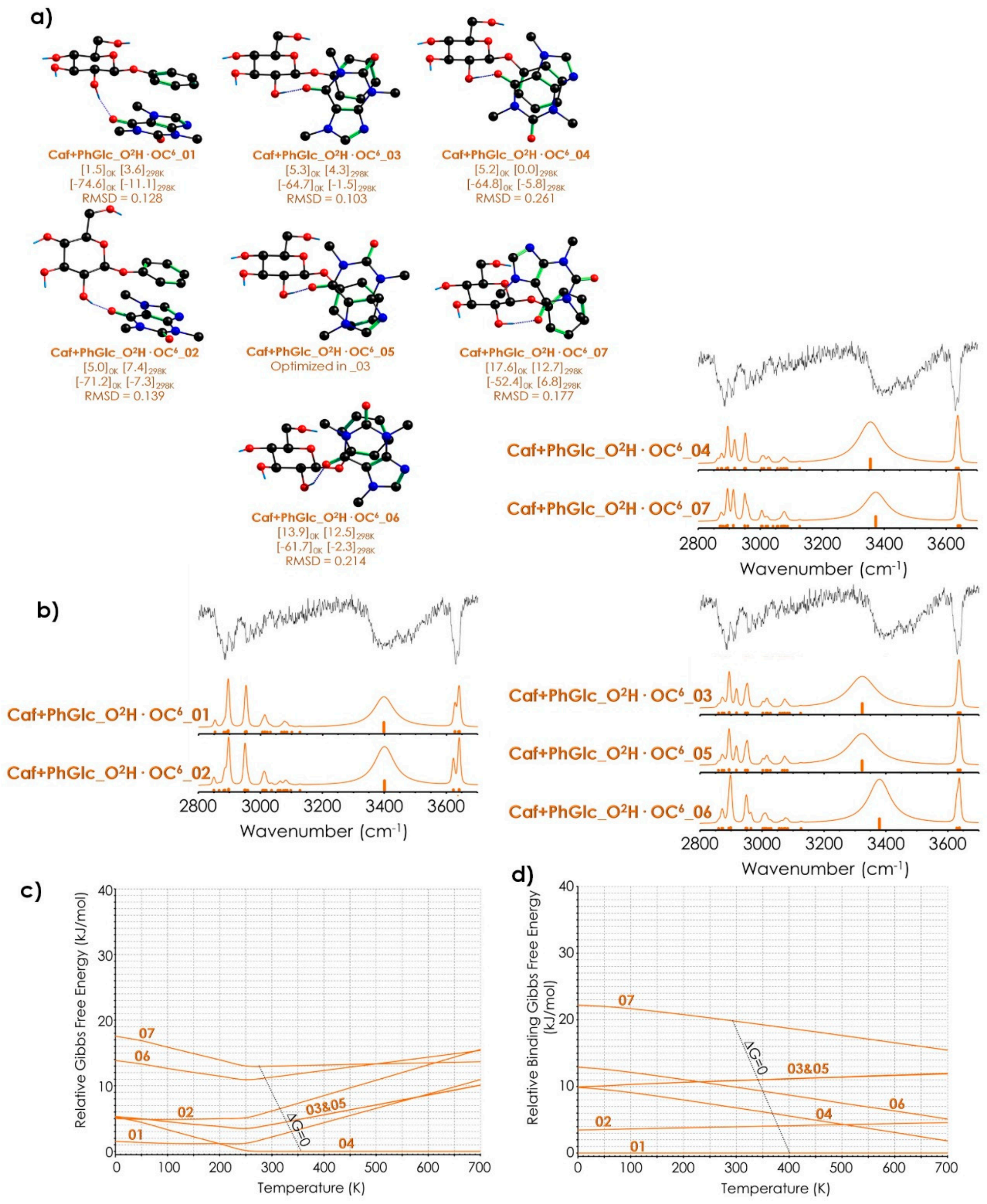

Figure S18: a) Complete set of structures of the $O^2H \cdot OC^2$ family, calculated at M06-2X/6-311++G(d,p) level. The first two values below each structure are the relative energy at 0 and 298 K, while the second pair of values correspond to the binding Gibbs free energy values; b) Comparison between the experimental spectrum (black trace) and the spectrum predicted for each conformation (green traces); c) Relative energy diagram calculated for the whole temperature range, referred to the global minimum; d) Relative binding Gibbs free energy diagram calculated for the whole temperature

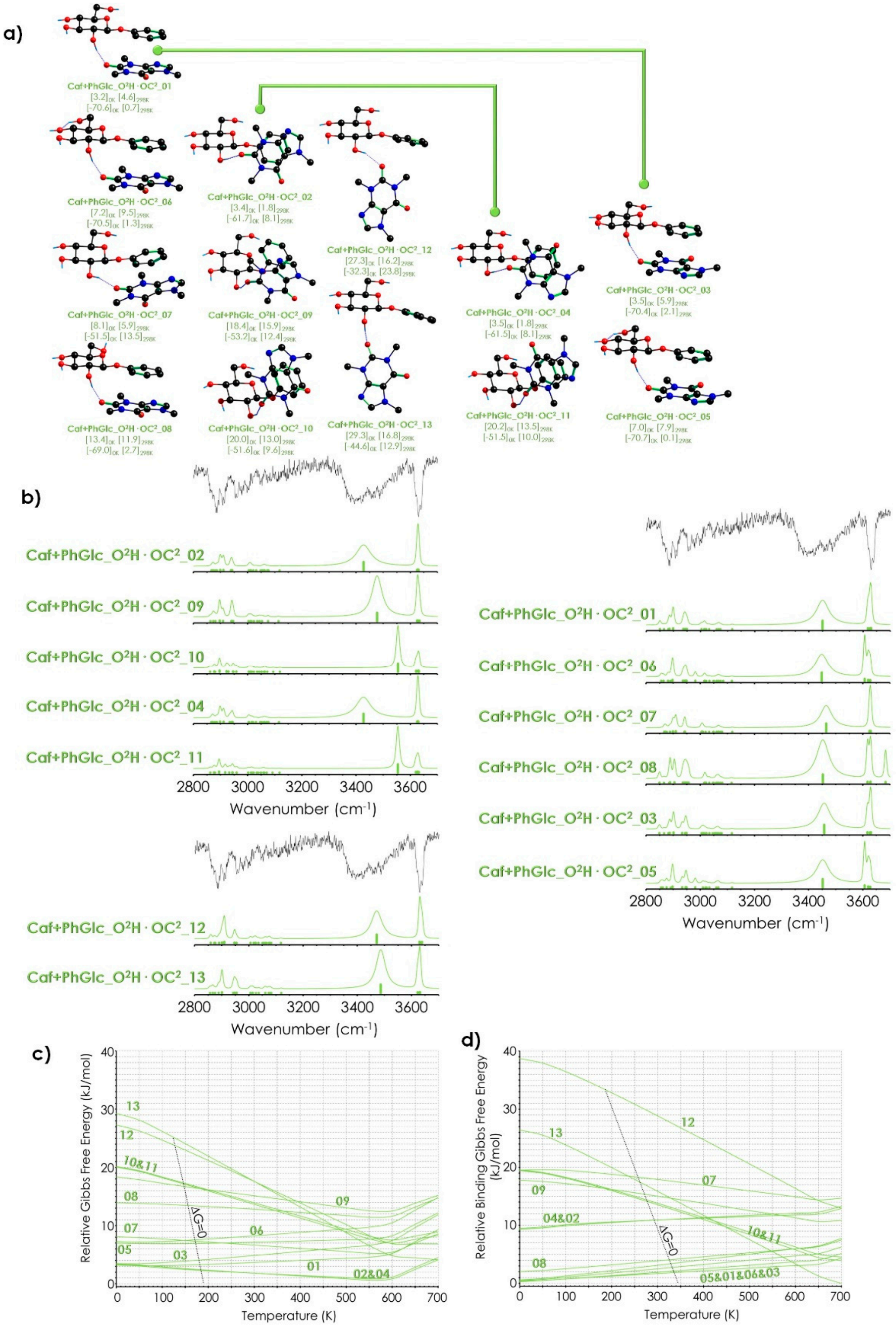

range.

Figure S19: a) Complete set of structures of the O²H · OC² family, calculated at B3LYP-GD3BJ/def2TZVP level. The first two values below each structure are the relative energy at 0 and 298 K, while the second pair of values correspond to the binding Gibbs free energy values; b) Comparison between the experimental spectrum (black trace) and the spectrum predicted for each conformation (green traces); c) Relative energy diagram calculated for the whole temperature range, referred to the global minimum; d) Relative binding Gibbs free energy diagram calculated for the whole temperature range

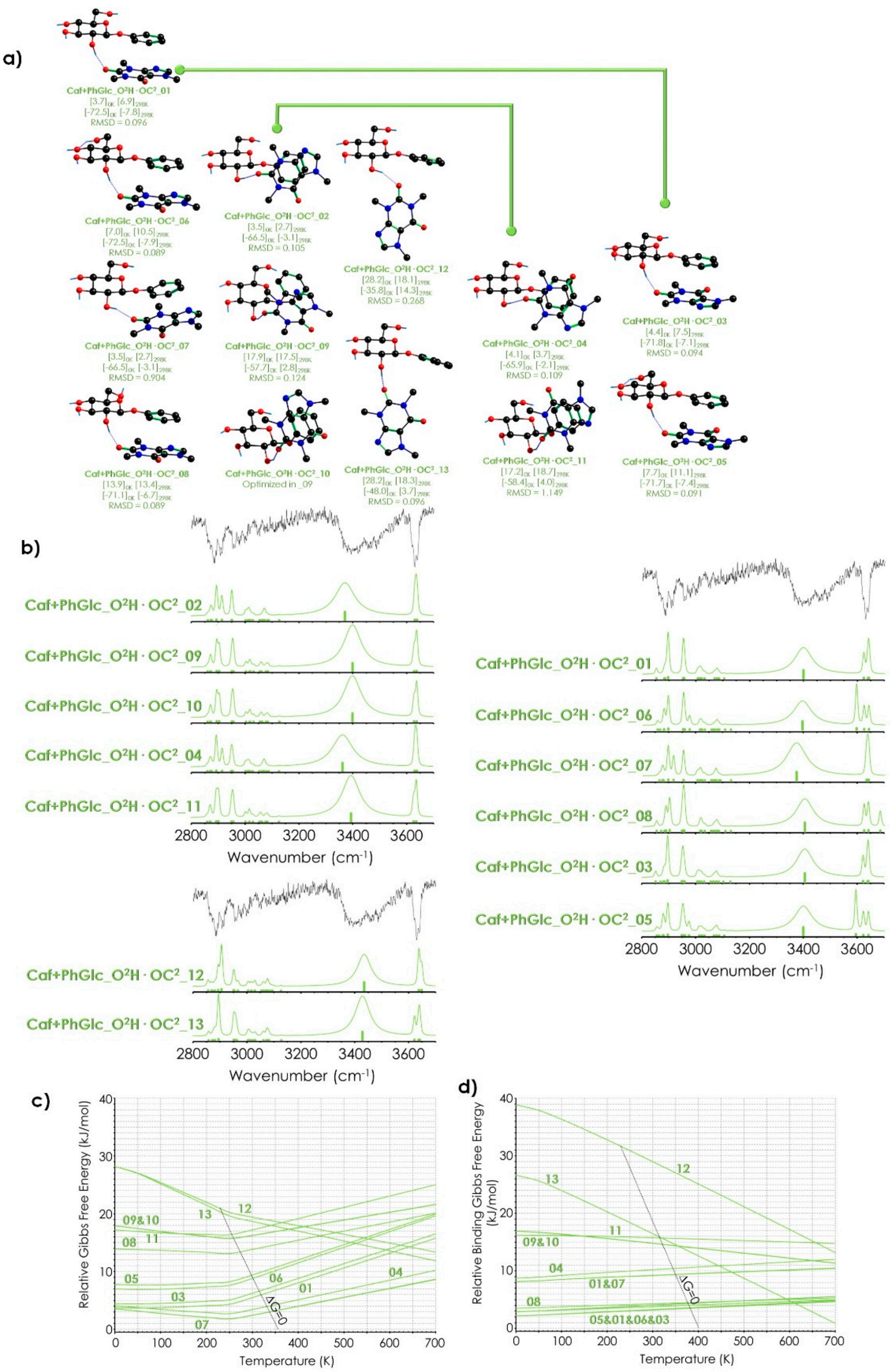

.

Figure S20: a) Complete set of structures of the O⁶H · N⁹ family, calculated at M06-2X/6-311++G(d,p) level. The first two values below each structure are the relative energy at 0 and 298 K, while the second pair of values correspond to the binding Gibbs free energy values; b) Comparison between the experimental spectrum (black trace) and the spectrum predicted for each conformation (orange traces); c) Relative energy diagram calculated for the whole temperature range, referred to the global minimum; d) Relative binding Gibbs free energy diagram calculated for the whole temperature range.

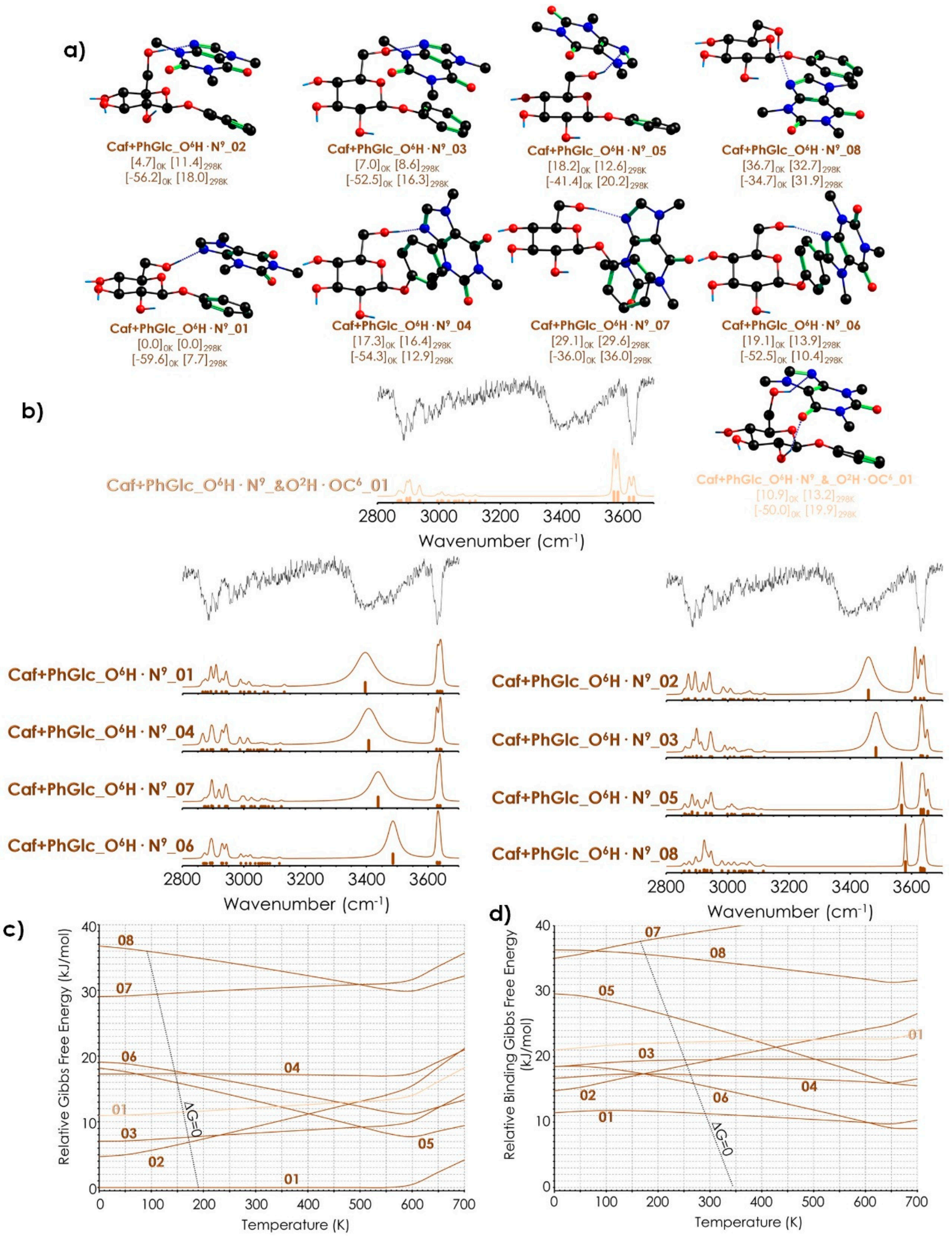

Figure S21: a) Complete set of structures of the O⁶H · N⁹ family, calculated at B3LYP-GD3BJ/def2TZVP level. The first two values below each structure are the relative energy at 0 and 298 K, while the second pair of values correspond to the binding Gibbs free energy values; b) Comparison between the experimental spectrum (black trace) and the spectrum predicted for each conformation (orange traces); c) Relative energy diagram calculated for the whole temperature range, referred to the global minimum; d) Relative binding Gibbs free energy diagram calculated for the whole temperature range.

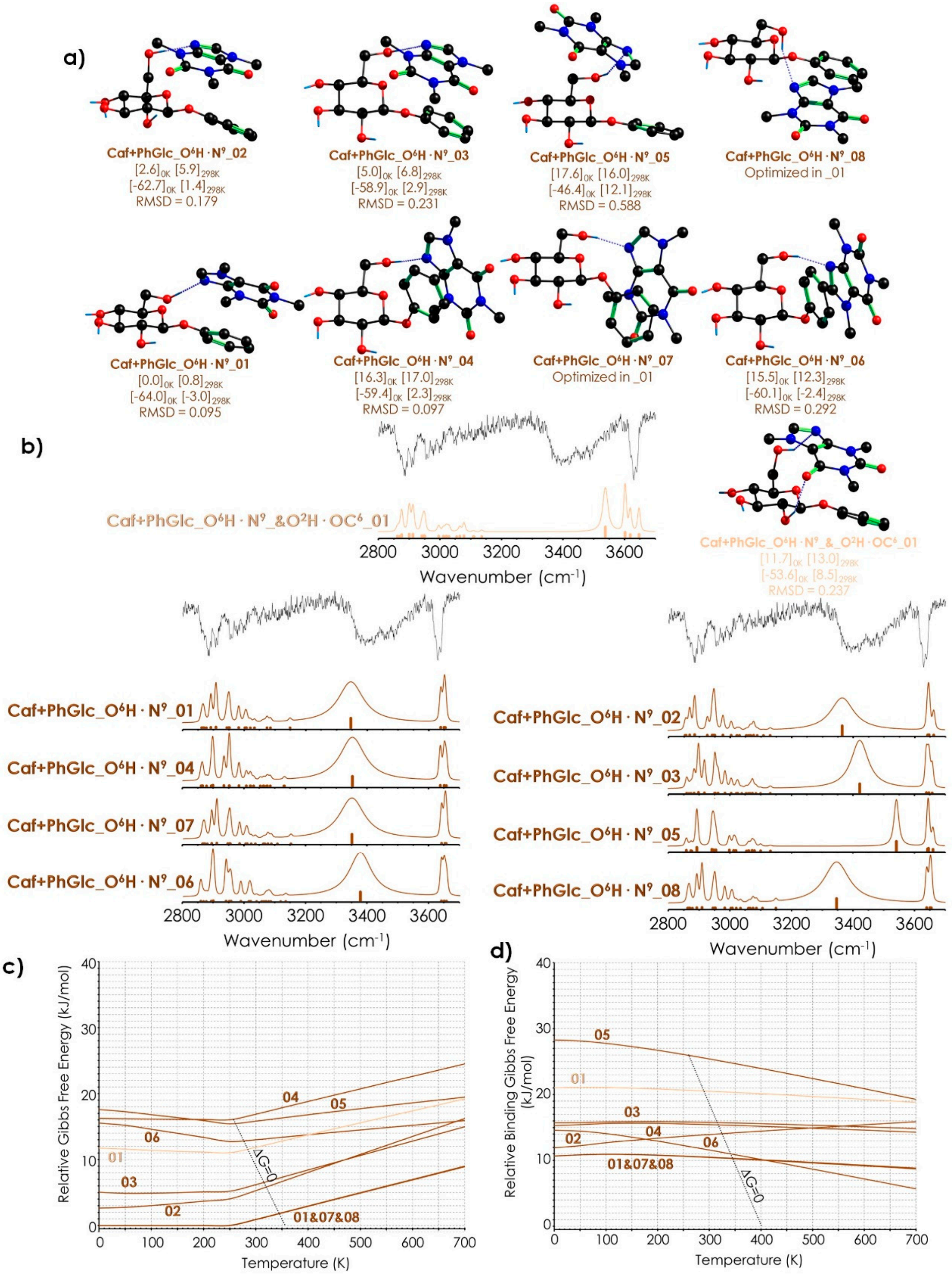

Figure S22: a) Complete set of structures of the O²H · N⁹ family, calculated at M06-2X/6-311++G(d,p) level. The first two values below each structure are the relative energy at 0 and 298 K, while the second pair of values correspond to the binding Gibbs free energy values; b) Comparison between the experimental spectrum (black trace) and the spectrum predicted for each conformation (dark green traces); c) Relative energy diagram calculated for the whole temperature range, referred to the global minimum; d) Relative binding Gibbs free energy diagram calculated for the whole temperature range.

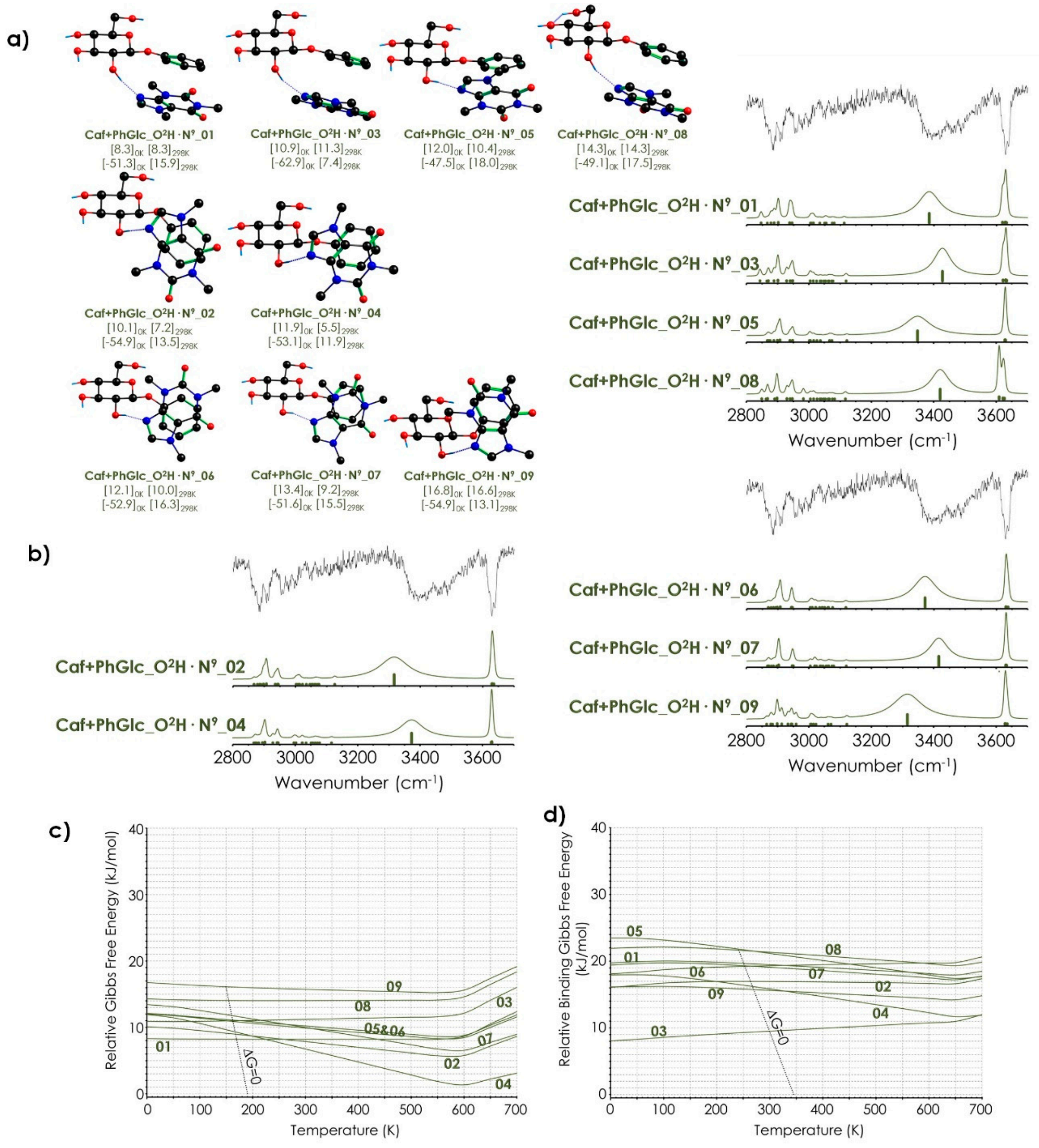

Figure S23: a) Complete set of structures of the O²H· N⁹ family, calculated at B3LYP-GD3BJ/def2TZVP level. The first two values below each structure are the relative energy at 0 and 298 K, while the second pair of values correspond to the binding Gibbs free energy values; b) Comparison between the experimental spectrum (black trace) and the spectrum predicted for each conformation (dark green traces); c) Relative energy diagram calculated for the whole temperature range, referred to the global minimum; d) Relative binding Gibbs free energy diagram calculated for the whole temperature range.

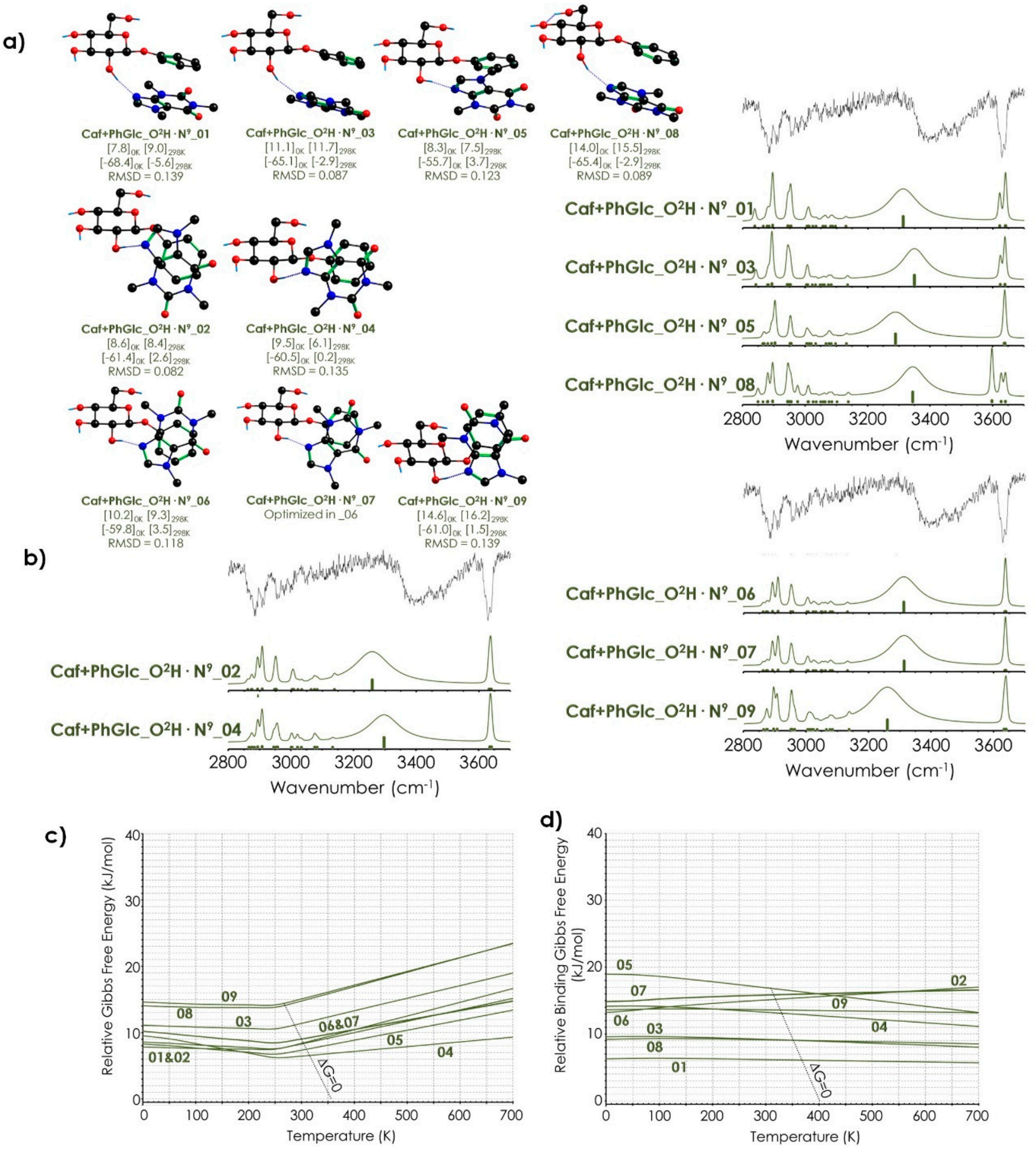

Figure S24: The most stable structures of the complex Caf+PhGlc were optimized using 5 different level of calculations: M06-2X/6-311++G(d,p), B3LYP-GD3BJ/6-311++G(d,p), B3LYP-GD3BJ/def2TZVP, B97D/6-311++G(d,p) and WB97X/6-311++G(d,p). Relative Binding Gibbs free energy of those structures at 0 K (top) and at 298 K (bottom panel).

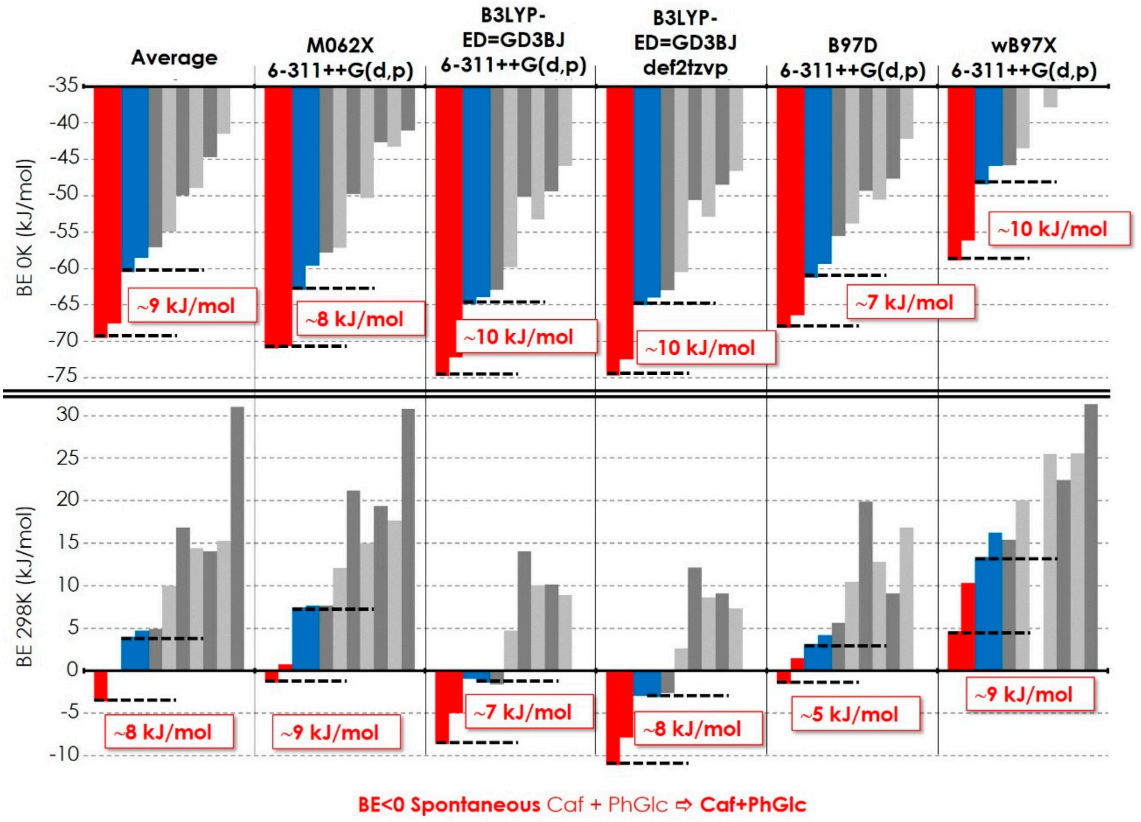

Figure S25: Gibbs free energy diagrams at M06-2X/6-311++G(d,p) (a, b) and B3LYP-GD3BJ/def2TZVP (c, d) of the most stable conformers of each family. a) and c) correspond to the relative Gibbs free energies, while b) and d) correspond to relative Binding Gibbs free energies.

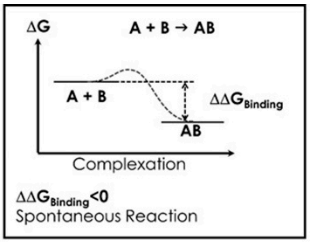

$$\Delta G_{A,0K} = E_A + ZPE$$

$$\Delta\Delta G_{AB,0K} = \Delta G_{AB,0K} - (\Delta G_{A,0K} + \Delta G_{B,0K})$$

$$\Delta G_{A,298K} = \Delta G_{A,0K} + Correction_{298K}$$

$$\Delta\Delta G_{AB,298K} = \Delta G_{AB,298K} - (\Delta G_{A,298K} + \Delta G_{B,298K})$$

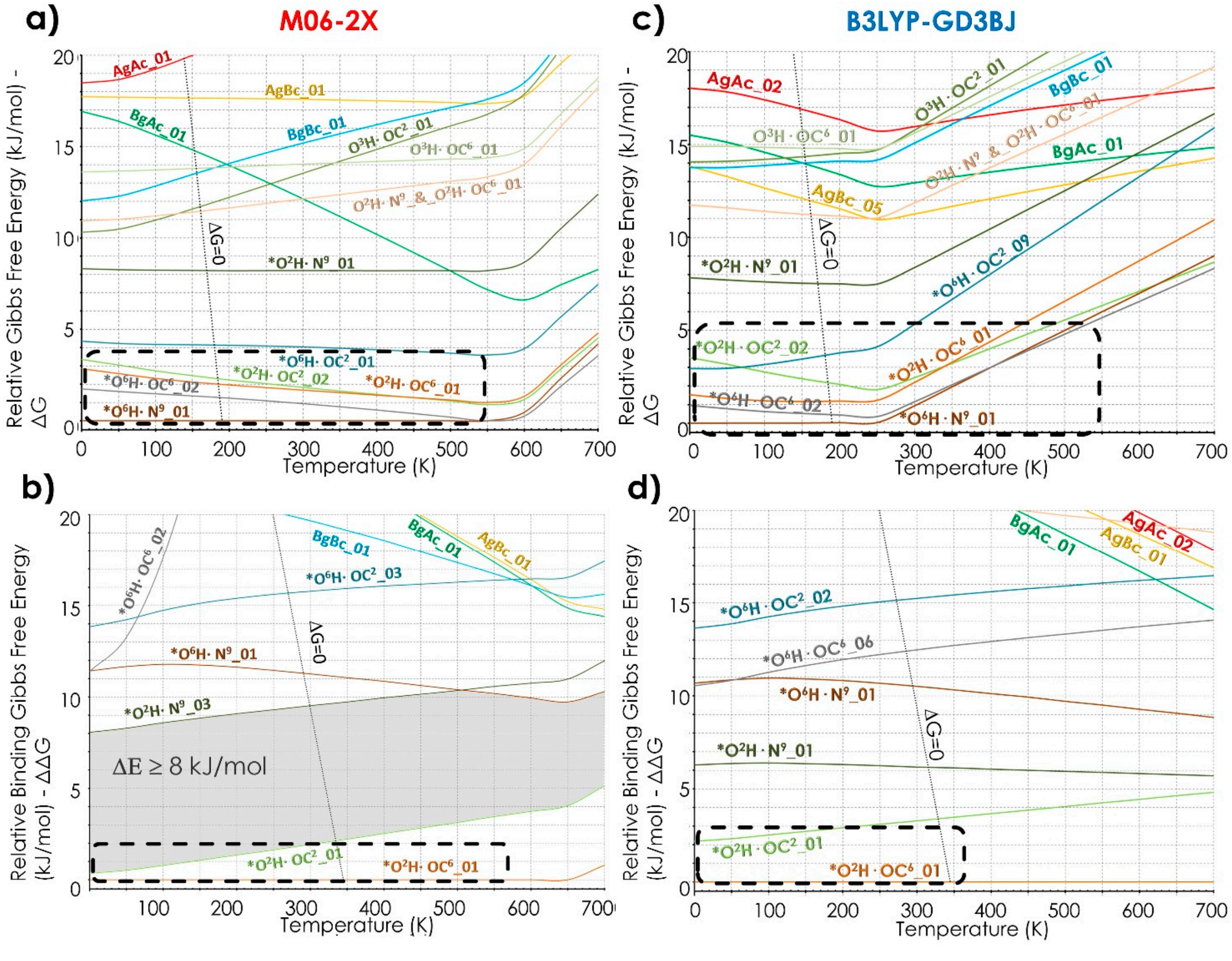

Figure S26: Experimental results. a) Resonant enhanced multi-photon ionization (REMPI) spectrum of the Caffeine+Phenyl-β-D-Glucopyranoside complex; b) REMPI spectrum of Phenyl-β-D-Glucopyranoside; c) REMPI spectrum of Caffeine. d) Ion dip infrared spectrum (IDIRS) of Caffeine+Phenyl-β-D-Glucopyranoside dimer, ionizing at 36670 cm^{-1}; e) IDIRS spectrum of Caffeine+Phenyl-β-D-Glucopyranoside complex, ionizing at 36916 cm^{-1}.

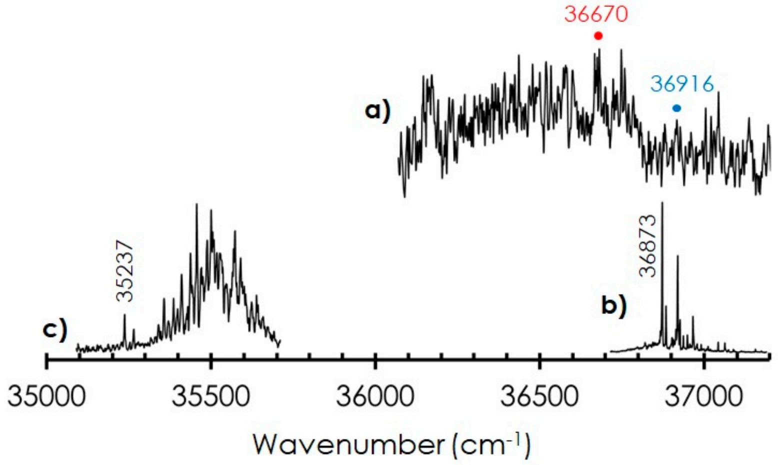

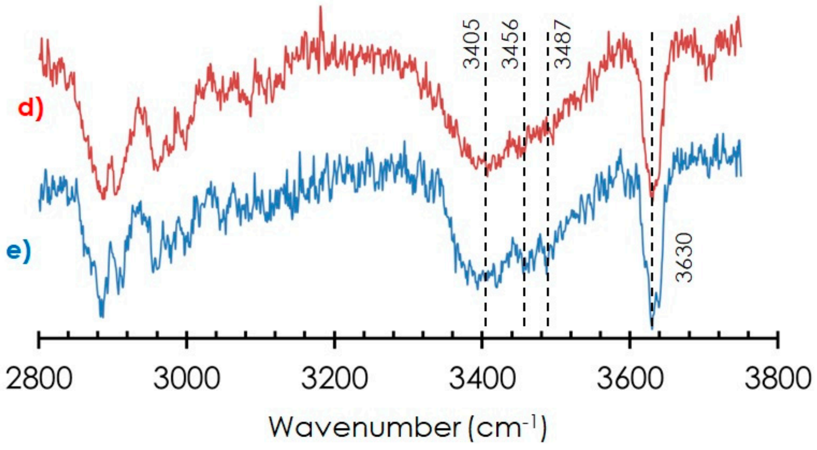

Figure S27: The average spectra of each family at M06-2X/6-311++G(d,p) (left) and B3LYP-GD3BJ/def2TZVP (right) theory levels.

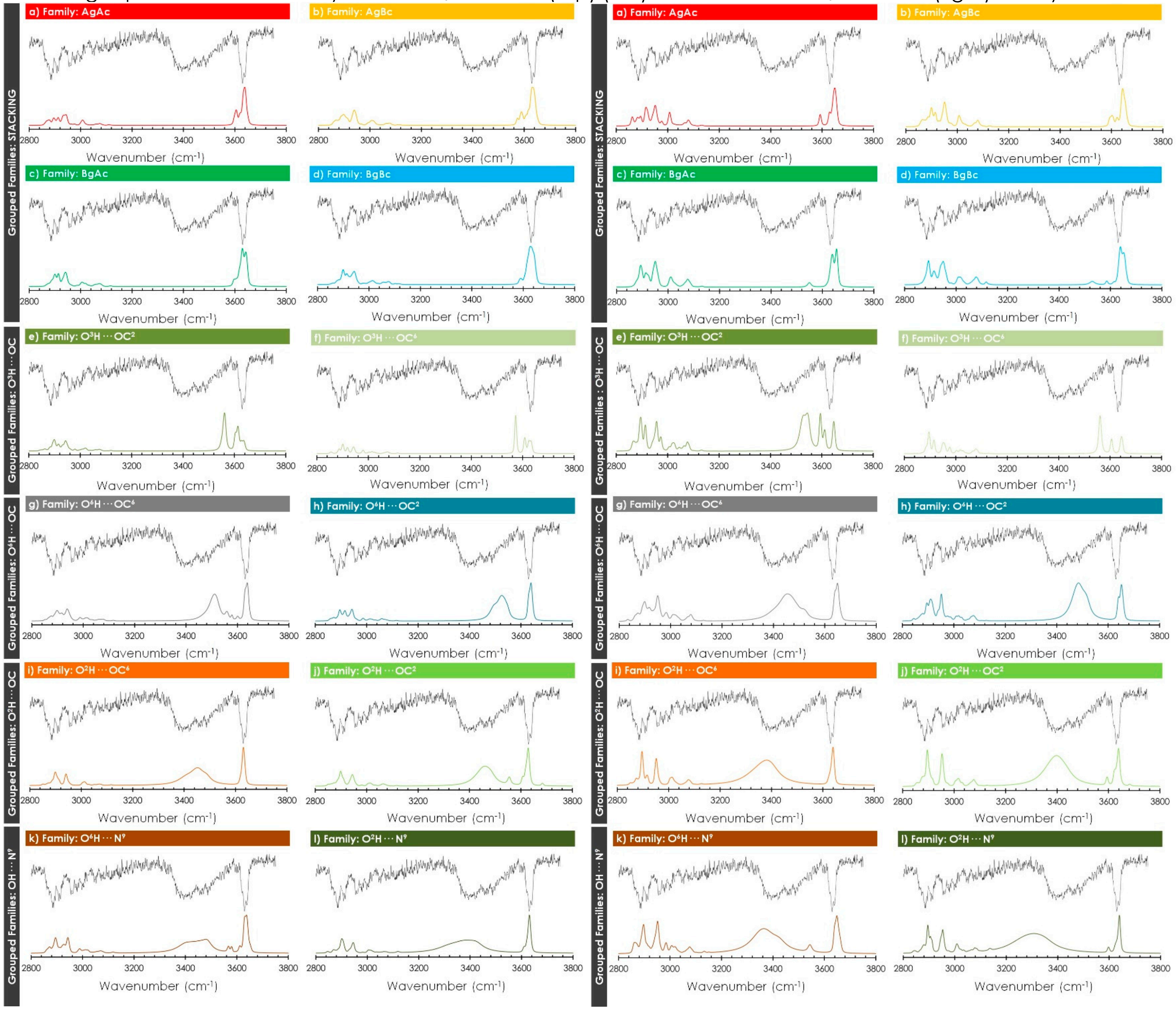

Figure S28: The average spectra of each group of families (5 groups of families) calculated at two theoretical levels.

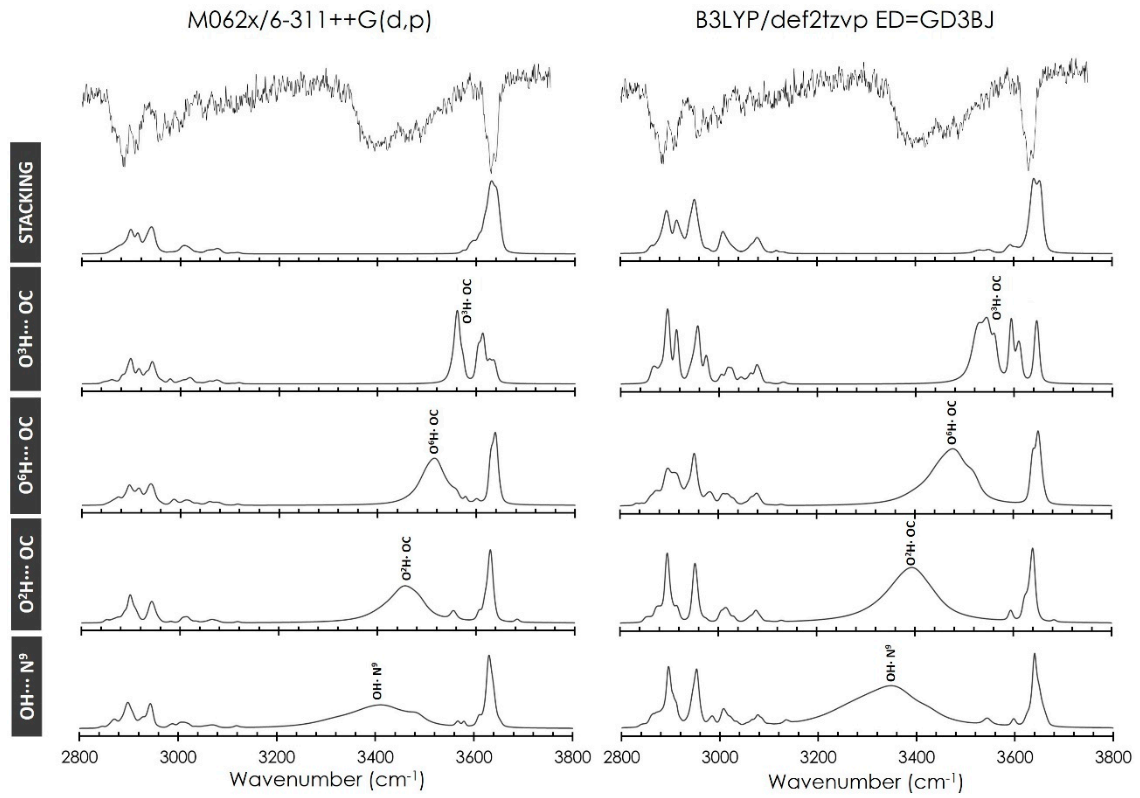

Figure S29: Data relative to Caf+Phenol complex.[6] On the left side, the energy diagram of the relative Gibbs free energy and the relative binding Gibbs free energy is reported. Since both molecules have only one conformer each, this makes that the two diagrams coincide. On the right side, the assignment of the experimental spectra (black traces) is shown.

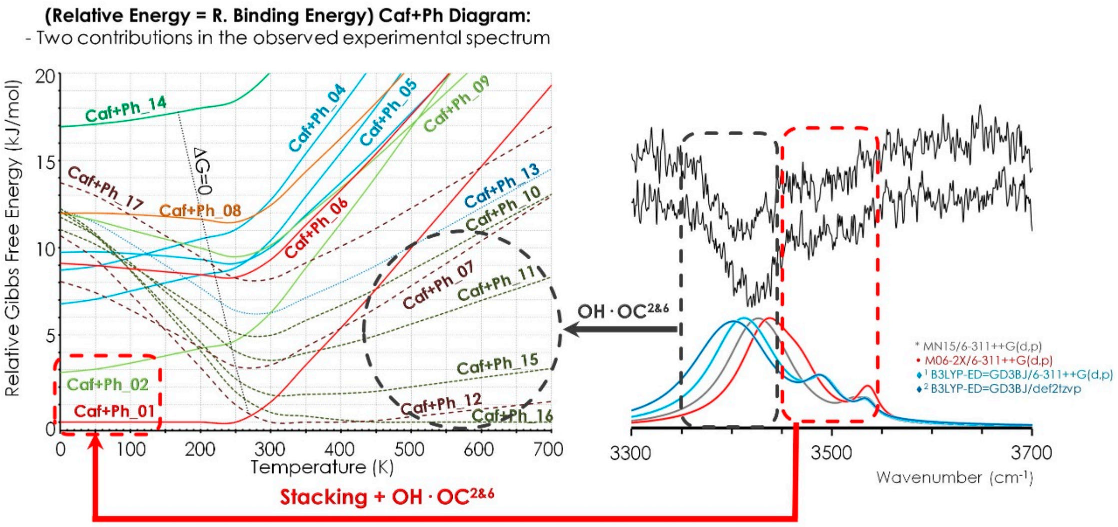

Figure S30: The most stable conformations of each family of Caf+PhGlc dimer. The interaction with the Ph moiety is omnipresent and for this reason, its position is compared using the root-mean-squared-deviation (RMSD, only taking into account carbon and oxygen atoms) with the corresponding equivalent structure of Caf+Ph complex. Below each structures, the binding Gibbs free energy at 0 K and at 298 K is given (kJ/mol).

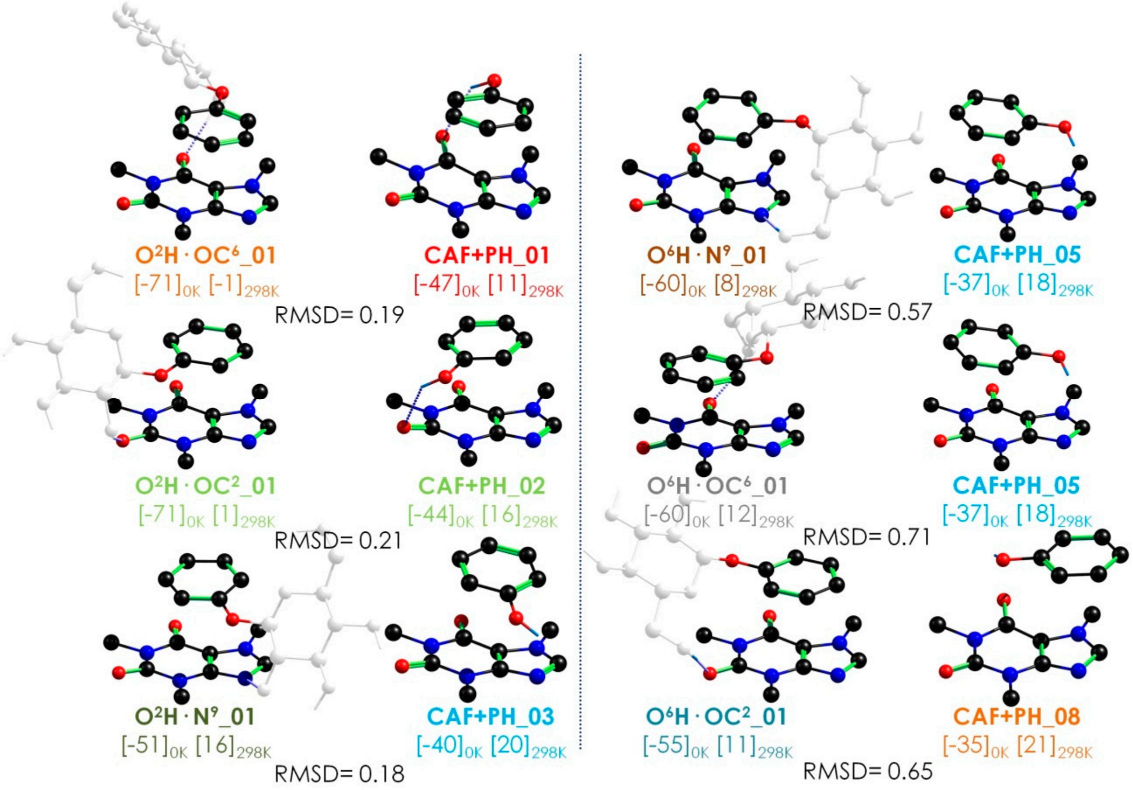

Figure S31: Comparison between the PDB crystal structures of caffeine inside the adenosine A_{2A} receptor (5MZP and 3RFM) and the different conformations of Caf+PhGlc.[4,5] The global minimum of Caf+PhGlc ($O^6H{\cdot}N^9$_01) does not correspond to the arrangement found in the crystal. Actually, the strongest bonded structures ($O^2H{\cdot}OC^2$_01 and $O^2H{\cdot}OC^6$_01) represent the best match.

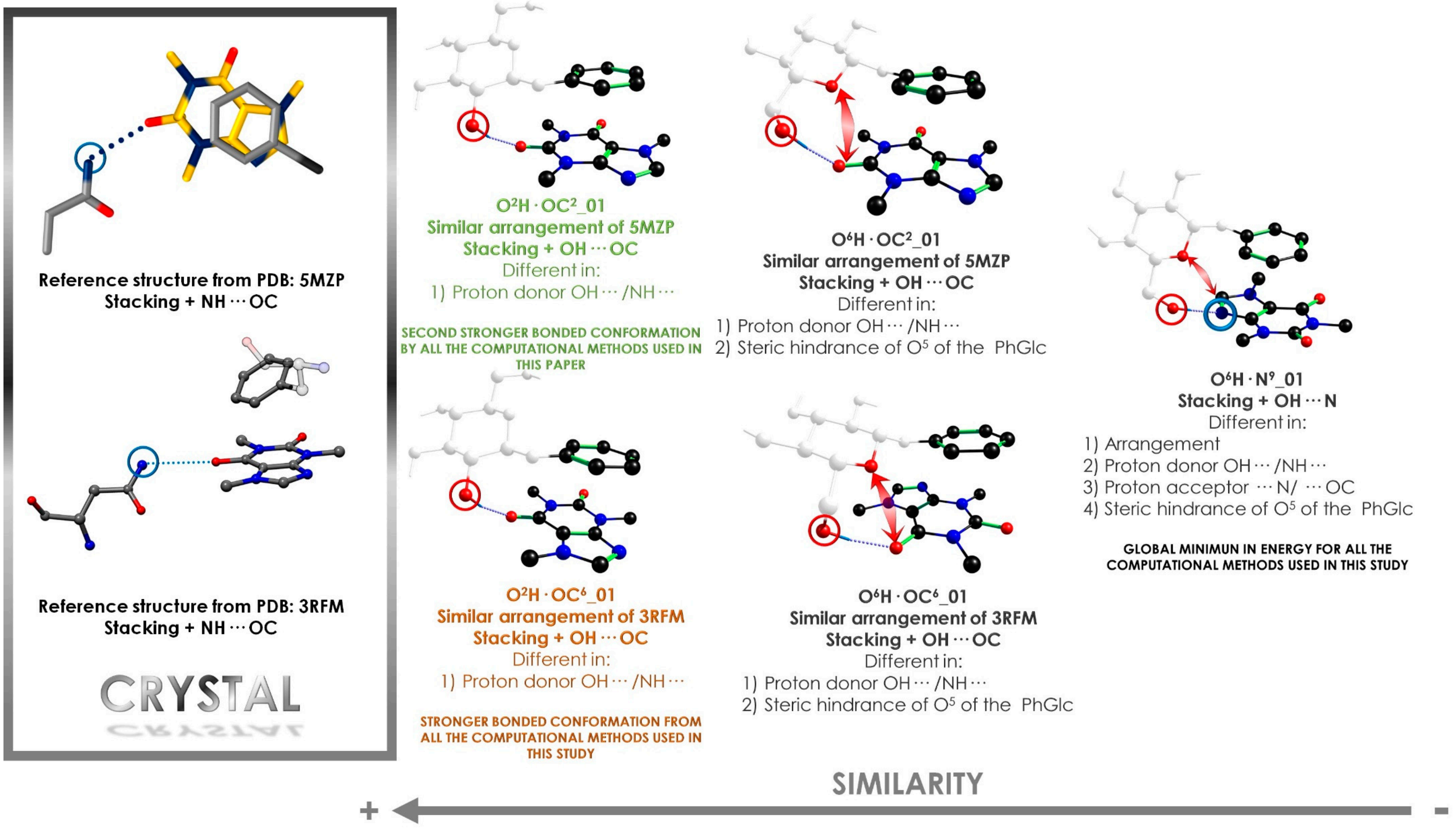

Figure S32: This diagram summarises the mechanism of a complexation reaction and thus the relative stability of the reactants and products. Caf, which has only one conformational isomer, reacts with two different isomers of PhGlc. The more stable isomer of PhGlc (in red) produces a conformational isomer of equivalent stability to the complex formed by Caf and the less stable isomer of PhGlc (in blue). Both isomer complexes are equivalent in terms of stability, but the subtracted binding energy in the blue complex (AB_2) is greater than the subtracted binding energy in the red complex (AB_1). As can be seen in the figure below, the AB_1 complex produces greater intramolecular stabilisation than the AB_2 complex. The AB_2 complex decreases the strength of intramolecular interactions to produce stronger intermolecular bonds.

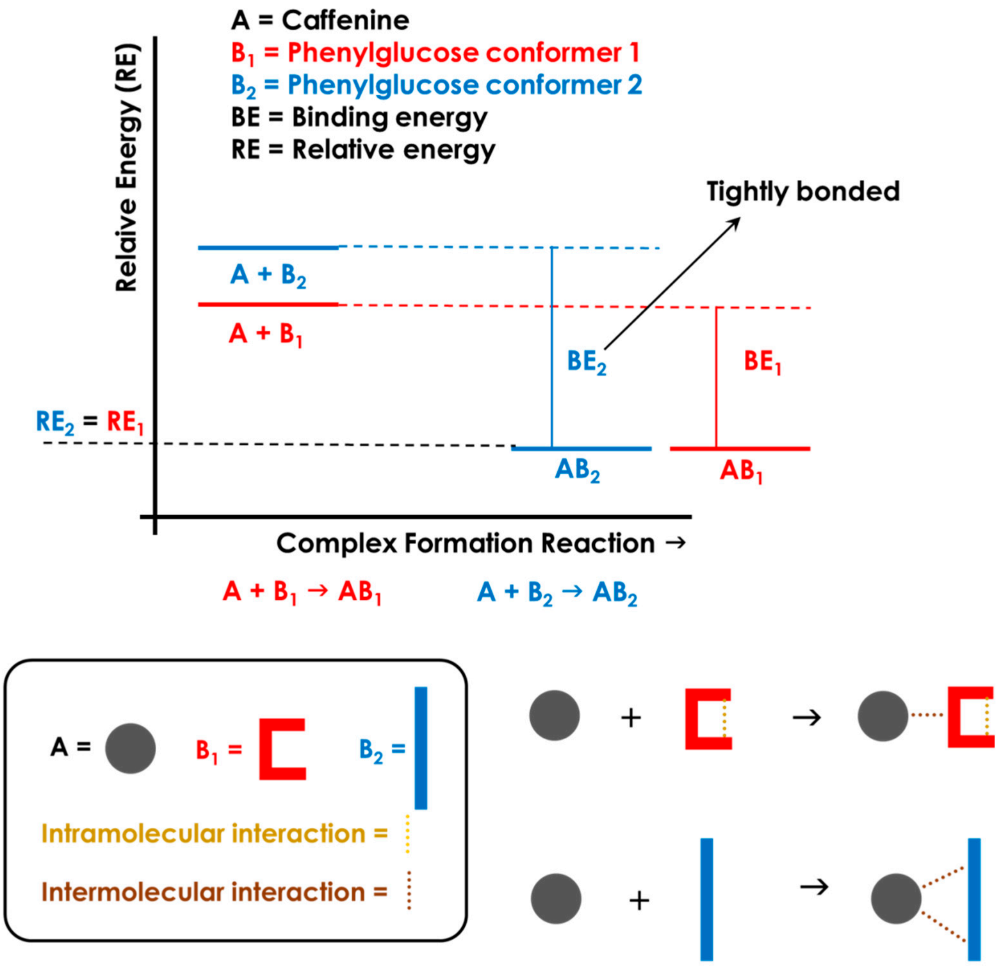

REFERENCES

1. Banks, J. L. *et al.* Integrated Modeling Program, Applied Chemical Theory (IMPACT). *J. Comput. Chem.* **26**, 1752–1780 (2005).

2. Halgren, T. A. Merck molecular force field. I. Basis, form, scope, parameterization, and performance of MMFF94. *J. Comput. Chem.* **17**, 490–519 (1996).

3. Case, D.A.; Ben-Shalom, I.Y.; Brozell, S.R.; Cerutti, D.S.; Cheatham, T.E.; Cruzeiro, V.W.D.; Darden, T.A.; Duke, R.E.; Ghoreishi, D.; Gilson, M.K.; Gohlke, H.; Goetz, A.W.; Greene, D.; Harris, R.; Homeyer, N.; Izadi, S.; Kovalenko, A.; Kurtzman, T.; Lee, P. A. AMBER 2018. *AMBER18* (2018).

4. Cheng, R. K. Y. *et al.* Structures of Human A1 and A2A Adenosine Receptors with Xanthines Reveal Determinants of Selectivity. *Structure* **25**, 1275-1285.e4 (2017).

5. Doré, A. S. *et al.* Structure of the adenosine A2A receptor in complex with ZM241385 and the xanthines XAC and caffeine. *Structure* **19**, 1283–1293 (2011).

6. Usabiaga, I., Camiruaga, A., Calabrese, C., Maris, A. & Fernández, J. A. Exploring Caffeine–Phenol Interactions by the Inseparable Duet of Experimental and Theoretical Data. *Chem. - A Eur. J.* **25**, 14230–14236 (2019).
